# A systematic review of structure–activity relationships in bacterial levan: physicochemical mediation and context-dependent functionality

**DOI:** 10.1039/d6ra04924a

**Published:** 2026-10-05

**Authors:** Tran Thi Thu Suong, Nguyen Phung Kieu, Hoang Ngoc Cuong, Tran Dinh Thang, Nguyen Ngoc Tuan

**Affiliations:** a Department of Food Technology, Institute of Biotechnology and Food Technology (IBF), Industrial University of Ho Chi Minh City (IUH) Ho Chi Minh City Vietnam; b Faculty of Food Technology, Binh Duong University (BDU) Ho Chi Minh City Vietnam; c Faculty of Health Sciences (FHS), Industrial University of Ho Chi Minh City (IUH) Ho Chi Minh City Vietnam nguyenngoctuan@iuh.edu.vn +84 938 759 525

## Abstract

Bacterial levan is a β-(2 → 6)-linked fructan with variable β-(2 → 1) branching, molar-mass distribution and higher-order organization. Although these descriptors are frequently associated with antioxidant, anti-inflammatory and prebiotic outcomes, the evidential basis for direct structure–activity relationships remains uncertain. We systematically reviewed 26 primary studies identified through Scopus and Web of Science Core Collection using a PRISMA 2020-informed process. Eligibility was separated from post-inclusion appraisal, and studies were classified according to whether they provided direct within-study evidence, indirect triangulated evidence or contextual observations only. Evidence was synthesized separately for chemical antioxidant assays, cell-based inflammatory models, microbial fermentation/prebiotic systems, gastrointestinal-protection models and material-performance tests. Across regimes, molar mass alone did not consistently explain activity, and few studies jointly measured polymer structure, physicochemical state and functional outcome. Diffusion, aggregation, molecular accessibility and interfacial interactions are therefore not treated as established mediators; rather, they form a testable interpretive model consistent with the available indirect evidence. Low-resolution studies were retained to characterize the evidentiary landscape but were excluded from positive mechanistic inference. This review defines the minimum analytical and experimental conditions required to test levan structure-function hypotheses and provides a chemistry-centred framework for future, within-system validation.

## Abstract 2

This systematic review shows that bacterial levan molar mass is not a universal activity determinant and proposes a testable, regime-specific structure-physicochemical state function model for future validation.

## Graphical abstract

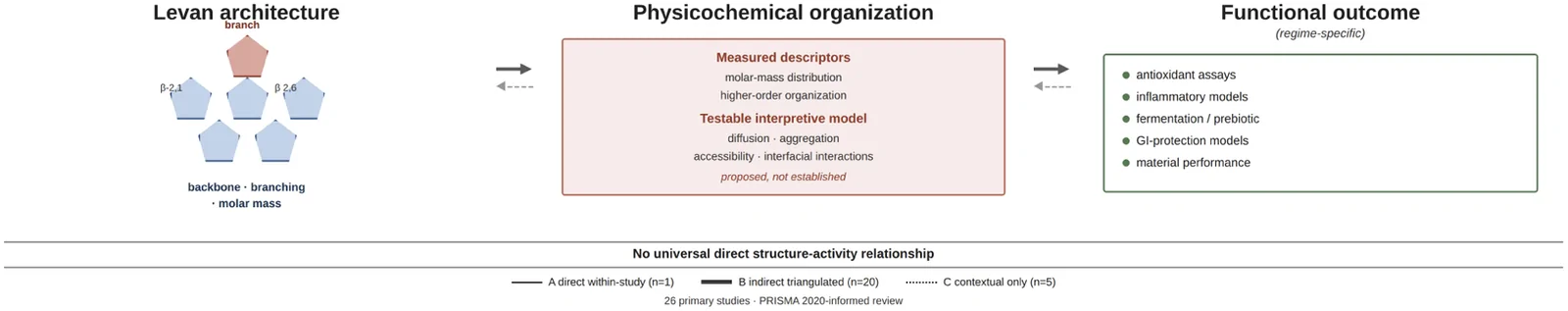

## 1. Introduction

Structure–activity relationships are widely employed to interpret the functional properties of bioactive compounds.^1–5^ However, when applied to polymeric systems such as bacterial levan, this framework becomes conceptually inadequate for mechanistic interpretation. Unlike small molecules, whose structure–function relationships can often be defined through discrete and comparable descriptors, polysaccharides exhibit multilevel organization in which molecular architecture, supramolecular assembly, and physicochemical behavior are intrinsically coupled.^3,4,6,7^ Under these conditions, the assumption that individual structural descriptors directly determine biological activity becomes increasingly untenable.

Despite this limitation, the current literature on bacterial levan continues to rely heavily on descriptor-based interpretations, in which parameters such as molar mass are treated as primary determinants of bioactivity.^8–10^ The resulting body of evidence appears highly inconsistent, with reported antioxidant, anti-inflammatory, and prebiotic effects varying substantially across studies.^11–14^ This inconsistency is typically attributed to biological variability; however, such an explanation remains insufficient. A closer examination suggests that the field has systematically conflated structurally interpretable evidence with context-dependent observations, leading to the construction of SAR patterns that lack mechanistic grounding.

In parallel, the global burden of chronic diseases including metabolic disorders, gastrointestinal dysfunction, and low-grade systemic inflammation has increased substantially, reinforcing the importance of diet-based strategies for long-term health modulation.^15–17^ These conditions are increasingly recognized as interconnected processes involving oxidative stress, immune dysregulation, and gut microbiota imbalance.^4,18,19^ Despite extensive research into functional foods and nutraceuticals, current dietary interventions often show limited consistency in delivering sustained physiological benefits.^20–22^ A key limitation lies in the persistent gap between *in vitro* bioactivity and *in vivo* nutritional functionality.^23,24^ Many compounds demonstrate promising antioxidant, anti-inflammatory, or prebiotic effects under controlled experimental conditions, yet fail to translate effectively within complex food matrices and biological systems.^25,26^ This discrepancy reflects not only biological variability but also a fundamental lack of integration of physicochemical behavior, digestion dynamics, and matrix interactions in the interpretation of bioactivity.

Within this context, natural polysaccharides have emerged as promising multifunctional components due to their structural diversity and their capacity to interact with multiple biological pathways.^2–4^ However, despite extensive investigation, their structure–activity relationships remain incompletely resolved and often inconsistent across studies.^1,27^ Unlike discrete molecular systems, polysaccharides exist as heterogeneous ensembles in which multiple structural dimensions co-vary and collectively influence functional behavior.^6,28,29^ Current SAR interpretations therefore often rely on simplified descriptors most notably molar mass treated as primary determinants of activity.^8,30^ Such approaches overlook physicochemical mediation, particularly diffusion, aggregation, and substrate accessibility which governs how structural attributes are expressed across experimental systems.^31,32^ In addition, the lack of standardization in structural characterization and the heterogeneity of assay platforms further limit the comparability of findings, hindering their translation into practical food and nutritional applications.

Bacterial levan provides a particularly relevant model system for addressing these challenges. As a β-(2 → 6)-linked fructan with variable β-(2 → 1) branching, levan combines a relatively conserved backbone with substantial variability in molar mass, branching architecture, and supramolecular organization.^29,33^ This structural duality makes levan an ideal system for examining how polymer architecture is translated into physicochemical behavior. Importantly, levan also exhibits properties directly relevant to food systems, including high solubility, viscosity modulation, water-binding capacity, and film-forming ability.^34,35^ These characteristics position it at the interface between structural biopolymers and functional food ingredients.^21,22,25^

At the same time, variability in microbial source, fermentation conditions, and downstream processing leads to a wide range of structural configurations,^29,30^ which are accompanied by equally disparate reports of antioxidant, anti-inflammatory, and prebiotic activities.^2,35^ These functional outcomes remain difficult to reconcile across studies, not only due to biological complexity but also because of the fragmented nature of structural characterization and the diversity of experimental contexts. Structural reporting remains uneven, with molar mass frequently documented, whereas branching architecture, dispersity, and higher-order organization are less consistently resolved.^27,28,33^ In parallel, biological outcomes are generated across heterogeneous assay systems that impose distinct physicochemical constraints, making direct comparison of results inherently problematic.^23,36^

These limitations reveal a fundamental conceptual issue: the application of descriptor-based SAR paradigms originally developed for small molecules to complex polymeric systems. In bacterial levan, structural attributes do not function as independent determinants of bioactivity. Instead, they influence functional outcomes indirectly through physicochemical mediation, including diffusion kinetics, aggregation states, and interfacial interactions.^29,32,37^ These physicochemical properties are, in turn, expressed through a specific biological interface – for example a chemical assay system, a cellular or tissue system, or the gut microbiota – such that a given structural feature can yield divergent functional readouts depending on which interface is engaged. These processes are expressed differentially across experimental and biological contexts, such that commonly reported descriptors most notably molar mass act as context-sensitive proxies rather than causal variables.

From a food science and nutrition perspective, this context dependency has profound implications for functional performance and nutritional interpretation.^21,22^ In real-world systems, levan is not encountered as an isolated molecular entity but as an integral part of a complex food matrix, in which its physicochemical properties govern both technological functionality and bioaccessibility.^21,38^ Factors such as dispersion states, interactions with co-existing macromolecules, and structural integrity during processing and gastrointestinal digestion dictate the extent to which levan interacts with microbial communities or biological targets.^25,26,39^ Consequently, functional outcomes observed under simplified *in vitro* conditions cannot be directly extrapolated to nutritional performance without accounting for these intervening physicochemical and matrix-dependent constraints.

Rather than attempting to establish direct structure–function relationships, this review addresses a fundamental question: under what conditions such relationships can be meaningfully interpreted in polymeric systems such as bacterial levan. In this context, reported biological activities are not treated as inherently comparable or structurally determined outcomes. Instead, the analysis evaluates how structural attributes are translated into physicochemical behaviors, and how these behaviors are subsequently expressed within specific experimental contexts. The contribution of this work is therefore not to derive predictive or universal SAR rules, but to clarify the interpretive conditions under which structure-related evidence supports mechanistically meaningful inference. By doing so, this review repositions bacterial levan bioactivity as a context-dependent phenomenon and delineates the epistemic limits of descriptor-based interpretation in the current literature.

Taken together, these considerations indicate that interpreting SAR in bacterial levan requires an integrated, mechanism-oriented perspective that connects polymer structure, physicochemical behavior, and context-dependent functional expression. Accordingly, this review critically re-examines the construction and interpretation of SAR in bacterial levan within the current literature. Using a PRISMA 2020-informed systematic approach,^40^ evidence from primary studies reporting both structural characterization and biological activity-specifically within antioxidant, anti-inflammatory, and prebiotic domains, is synthesized.^13,41,42^ Rather than treating reported activities as inherently comparable, the analysis evaluates structure–function relationships in relation to structural resolution and experimental context, thereby constraining interpretation to mechanistically meaningful conditions.

This systematic review asks when reported associations between bacterial levan structure and function are mechanistically interpretable. Its objectives are to: (i) map the structural attributes and analytical methods reported for bacterial levan; (ii) evaluate evidence within distinct experimental regimes; and (iii) formulate a testable model linking polymer architecture, physicochemical state and functional readout. The model is not presented as proof of mediation. Direct evidence is reserved for studies that measure structure, a candidate physicochemical mediator and function within the same controlled system; cross-study consistency is treated as indirect evidence; and untested connections are identified as hypotheses.

## 2. Methodological framework

### 2.1. Search logic and role of PRISMA

Fig. 1 testable interpretive model linking bacterial levan structure to functional outcomes. Polymer structure constrains emergent physicochemical behavior (diffusion, aggregation, conformational flexibility, substrate accessibility, interfacial interaction), which is proposed to mediate functional expression (antioxidant, anti-inflammatory, prebiotic) through a biological interface (chemical assay, cellular/tissue system, or gut microbiota) specific to the experimental regime. Experimental context (top) and food-matrix/processing/digestion context (bottom) act as effect modifiers on this pathway, not as evidence of mediation. The structure-physicochemical-interface-outcome pathway is depicted as a single candidate line, reflecting that mediation has not been experimentally tested within any one system; arrow style is therefore uniform across all links and does not encode evidence strength. The evidence base supporting this model (Table 1; *n* = 26 studies) is instead summarized separately as a per-study Tier A/B/C distribution (Tier A, direct within-study support, *n* = 1; Tier B, indirect/triangulated support, *n* = 20; Tier C, minimal/contextual characterization, *n* = 5) and is not assigned to any individual arrow or link. This figure is therefore a data-grounded summary of the evidence landscape across the reviewed literature, not a claim that any single structure–function link is mechanistically proven.

**Fig. 1:**
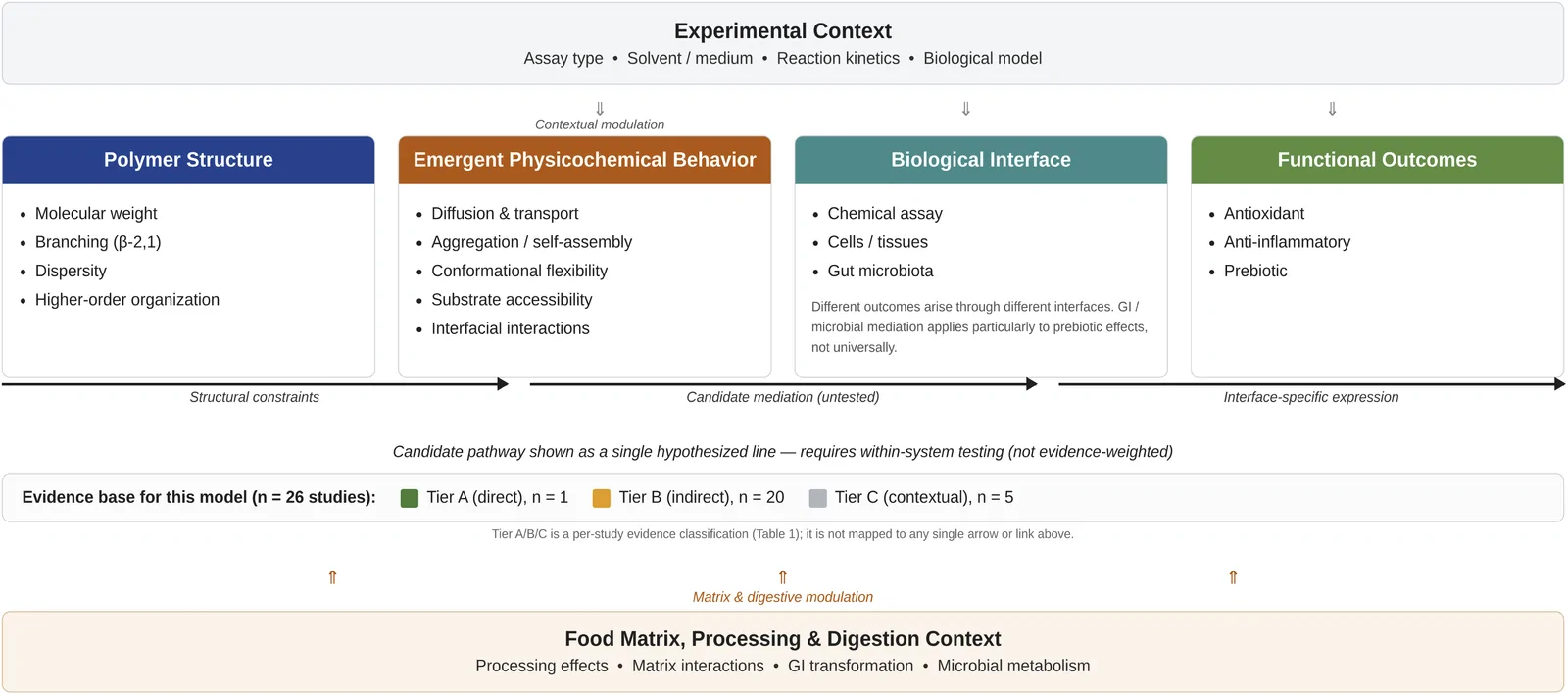
Testable interpretive model for bacterial levan structure–function relationships.

**Table 1:** Evidence-integration tiers (Tier A, B, C) for the 26 included studies, based on structural confirmation, physicochemical-mediator. Measurement, and comparator/co-variation control (*n* = 1 Tier A, 20 Tier B, 5 Tier C). Full per-study structural and mediator evidence, and the internal basis for each tier assignment, are given in SI Table S2^a^

Study (author, year)	Bacterial source (organism/strain)	Structural confirmation	Molar-mass method/reliability	Branching resolution	Physicochemical state measured (Y/N; method)	Experimental regime	Comparator/control (co-variation control)	Evidence-integration tier
Kim *et al.*, 2005 (ref. 48)	*Zymomonas mobilis* (ZM1)	High (^13^C-NMR; TEM; particle size)	2250 kDa (SEC/GPC (LS + viscometer); absolute)	NR	Yes; self-assembled nanoparticles (170-300 nm; amphiphilic self-assembly), TEM/particle size	Cell-based inflammatory	No	B
Abdel-Fattah *et al.*, 2012 (ref. 49)	*Bacillus subtilis* (NRC1aza)	Low (paper chromatography; HPLC; acid hydrolysis)	85.23; 31.95 kDa (viscometry; proxy)	NR	No	Chemical antioxidant	NR	C
Huang MeiYing *et al.*, 2013 (ref. 50)	*Bacillus licheniformis* (FRI MY-55)	Low (NR)	13.45 (range 13.45 → 2.96) kDa (NR; not reliable)	NR	No	Not clearly assignable (NR)	NR	C
Esawy *et al.*, 2013 (ref. 51)	*Bacillus subtilis* (M)	Moderate (^1^H/^13^C NMR, FTIR, chromatography)	21.8; 13.118; 9.53 kDa (viscometry; proxy)	NR	No	Chemical antioxidant	NR	B
Srikanth *et al.*, 2015 (ref. 11)	*Acetobacter xylinum* (NCIM 2526)	Moderate (FTIR; ^1^H/^13^C NMR; TGA; HPLC/sugar)	NR kDa (NR; not reliable)	Qualitative (branched; not quantified)	No	Cell-based inflammatory; chemical antioxidant	NR	B
Szwengiel & Nkongha, 2019 (ref. 52)	*Bacillus subtilis* (DSM 347)	High (GPC (TDA/CC); UHPLC-MS)	29.8; 2.3 kDa (GPC-SEC TDA (absolute mass); absolute)	NR	Yes (supportive only); size/compactness rationale (rheology not directly measured)	Microbial fermentation/prebiotic	No	B
Domżał-Kędzia *et al.*, 2019 (ref. 53)	*Bacillus subtilis natto* (KB1)	Moderate (FTIR; ^1^H/^13^C NMR; SEM; TGA; GPC)	37.62 kDa (SEC/GPC (triple detection); relative)	Low/qualitative (weak branching signals)	No	Chemical antioxidant	NR	B
Bouallegue *et al.*, 2020 (ref. 65)	*Bacillus subtilis* (AF17)	High (methylation (GC-MS); 1D/2D NMR; SEC-TDA)	20 000 kDa (HP-SEC-TDA; absolute)	Low (linear:branched ≈ 20 : 1; ∼5%)	Yes; aggregation state assessed *via* pH-dependent checks	Chemical antioxidant	Yes (explicit pH-dependent aggregation control)	B
Pei *et al.*, 2020 (ref. 41)	*Bacillus megaterium* (PFY-147)	Moderate (FTIR; ^1^H/^13^C NMR; SEM; AFM; HPLC)	1878 kDa (GPC; relative)	NR	Yes; porous/compact microstructure (SEM/AFM)	Chemical antioxidant	No – co-varies with MW and composition (uncontrolled)	B
Ragab *et al.*, 2020 (ref. 12)	*Bacillus* sp. (honey isolate)	Moderate (SEM; XRD; ^1^H/^13^C NMR; chromatography)	21 kDa (viscometry; proxy)	NR	Yes; film forming/adhesive property (protective coating mechanism)	Gastrointestinal-protection; microbial fermentation/prebiotic; cell-based inflammatory	Partial – mechanistic pathway explicitly modelled (NF-kB/COX-2)	B
María *et al.*, 2021 (ref. 55)	*Bacillus* sp. (4A)	Low (FTIR; HPLC (monosaccharide after hydrolysis))	NR kDa (NR; not reliable)	NR	Yes; emulsifying/hydrogel-forming behaviour	Microbial fermentation/prebiotic	No	C
Vu *et al.*, 2021 (ref. 56)	*Bacillus velezensis* (VTX20)	Moderate (^1^H/^13^C (HMBC) NMR; HPLC; genome mining)	NR kDa (NR; not reliable)	NR	No	Chemical antioxidant	NR	B
Koşarsoy Ağçeli, 2022 (ref. 57)	*Pseudomonas mandelii* (NR)	Low (FTIR; SEM; TGA)	NR kDa (NR; not reliable)	NR	Yes; composite/material film matrix (porosity/hydrophilicity implied)	Material/film performance; chemical antioxidant	No	C
Veerapandian *et al.*, 2023 (ref. 58)	*Bacillus subtilis* (MTCC 441)	Moderate (^1^H/^13^C NMR; GPC)	2831.2 kDa (GPC; relative)	NR	No	Chemical antioxidant	NR	B
Altıntaş *et al.*, 2023 (ref. 13)	*Halomonas elongata* (153B)	High (FTIR; 1H NMR; MALLS-GPC)	3260 kDa (MALLS-GPC; absolute)	NR	No	Material/film performance; microbial fermentation/prebiotic; cell-based inflammatory; chemical antioxidant	NR	B
Marimuthu & Rajendran, 2023 (ref. 59)	*Bacillus* sp. (EPS003)	Moderate (TLC; FTIR; 1H NMR; FE-SEM; XRD; EDAX; TGA)	NR kDa (NR; not reliable)	NR	Yes; porosity/hydrophilic matrix (FE-SEM/XRD/EDAX)	Chemical antioxidant	No	B
Saleh *et al.*, 2023 (ref. 60)	*Pseudomonas aeruginosa* (HI1)	Moderate (TLC; HPLC; FTIR; 1H/13C NMR)	NR kDa (NR; not reliable)	NR	No	Gastrointestinal-protection; microbial fermentation/prebiotic; cell-based inflammatory; chemical antioxidant	NR	B
Tilwani *et al.*, 2023 (ref. 38)	*Enterococcus faecium* (MC-5)	Moderate (HPLC; NMR; SEM; Congo red; XRD; FTIR)	24 kDa (SEC (dextran standards); relative)	NR	Yes; Congo red conformation assay (ordered/triple-helix) + film matrix	Chemical antioxidant	No	B
Thomas *et al.*, 2024 (ref. 10)	*Bacillus subtilis* (PR-C18)	Moderate (FTIR; 1H/13C NMR; SEM; EDX)	34 kDa (SEC/GPC (standards); relative)	NR	Yes; porous morphology (SEM) linked to antioxidant + prebiotic readouts	Material/film performance; microbial fermentation/prebiotic; chemical antioxidant	No	B
Mehta *et al.*, 2024 (ref. 61)	*Bacillus megaterium* (KM3)	Low (HPLC; FTIR; XRD; DSC/TGA; Congo red)	NR kDa (NR; not reliable)	NR	Yes; Congo red conformation assay (triple-helix)	Chemical antioxidant	No	C
Grinev *et al.*, 2024 (ref. 37)	*Paenibacillus polymyxa* (88A)	High (HPAEC-PAD; FTIR/DRIFT; 1D/2D NMR; SEM; AFM; TGA/DSC; rheology)	1900 kDa (HPGPC (standards); relative)	Slightly branched (qualitative)	Yes; porous/compact microstructure (SEM/AFM)	Chemical antioxidant	No – co-varies with MW and composition (sulfate groups noted)	B
Wahab *et al.*, 2024 (ref. 14)	*Bacillus megaterium* + *Rhizobium* sp*.* (coculture) (OP315322/OP363928)	Moderate (TLC; FTIR; ^1^H/^13^C NMR)	11.5 kDa (viscometry; proxy)	NR	No	Microbial fermentation/prebiotic; cell-based inflammatory; chemical antioxidant	NR	B
Li *et al.*, 2025 (ref. 42)	*Bacillus amyloliquefaciens* (JM033)	High (HPAEC-PAD; FTIR; methylation (GC-MS); 1D/2D NMR; SEC-MALLS)	17.6 kDa (SEC-MALLS-RI; absolute)	Branched (reported; not single % value)	No	Not clearly assignable (NR)	NR	B
Yan *et al.*, 2025 (ref. 30)	*Bacillus licheniformis* (SFLV-ZM107)	High (FTIR; GPC; SEM; AFM; TGA; rheology)	7.655; 25.76 kDa (GPC-MALLS; absolute)	NR	Yes; rheology/viscosity and chain entanglement, LMW *vs.* HMW fraction comparison	Microbial fermentation/prebiotic	Yes – direct LMW-vs-HMW fraction comparison	A
Hata *et al.*, 2024 (ref. 9)	*Gluconobacter cerinus* (UELBM11)	High (methylation (GC-MS); FTIR; 1H/13C NMR; SEM; TGA/DTG)	878 kDa (HPSEC-MALLS; absolute)	Branched (5.87% branching points)	Yes; porous/compact microstructure (SEM)	Chemical antioxidant	No – co-varies with MW and composition (uncontrolled)	B
Wannasutta *et al.*, 2025 (ref. 35)	*Bacillus siamensis* (KKSB4 (+ other strains))	Moderate (TLC; FTIR; 1H/13C NMR; DSC)	61 000; 220 000; 85 000; 69 000 (apparent, DLS) kDa (DLS/Zetasizer (hydrodynamic; apparent, not absolute); proxy)	NR	Yes; UHMW *via* DLS/Zetasizer (method limitation) + WHC/OHC claims	Microbial fermentation/prebiotic; chemical antioxidant	No (method itself is non-standard for MW)	B

a Tier assignment follows the decision rule in Section 2.3 and SI Table S1. The superscript number after each study citation corresponds to the reference list (SI). ‘Physicochemical state measured’ indicates whether a candidate mediator was measured in the same study as the reported functional outcome. ‘Experimental regime’ classifies each study by its predominant experimental system or assay context (*e.g.*, chemical, cell-based, fermentation, gastrointestinal, or material/film-based); it is distinct from ‘biological activity domain’ (Table 2 and 3), which remains confined to the three domains defining eligibility (Section 2.2: antioxidant, anti-inflammatory, prebiotic). Gastrointestinal-protection and material/film-performance regime labels therefore describe the assay system within which one of these three domains was measured, not an additional eligibility domain. Two studies (ref. 42 and 50) could not be assigned to a single predefined regime and are reported as ‘Not clearly assignable (NR)’ here, with the underlying reasons given in Section 3.9 (‘Multi-regime studies and the two unclassified studies’)—this classification is independent of, and does not alter, the evidence-integration tier decision rule above.

The identification and selection of studies were guided by the PRISMA 2020 framework, which was applied as a procedural tool to ensure transparency and traceability in study identification and screening, rather than as a prescriptive structure for quantitative synthesis.^40,43^ In this study, PRISMA was used to document how studies were identified, screened, and retained, while the analytical emphasis was placed on the interpretive evaluation of evidence in relation to structure–activity relationships.

The literature search was conducted in Scopus (16 August 2025, 10:03–10:56 UTC) and Web of Science Core Collection (16 August 2025, 11:22–11:27 UTC). Search terms were iteratively refined to capture studies at the intersection of levan structure, physicochemical characterization, and experimentally evaluated biological activity,^27,44^ following PRISMA-S recommendations.^45^ Three progressively refined search strings (Tier 1–3) were executed separately in Scopus and Web of Science Core Collection, applied to the Title, Abstract, and Keywords fields, combining levan/fructan-related vocabulary with structural descriptors (*e.g.*, molar mass, branching, glycosidic linkage) and target bioactivities (antioxidant, anti-inflammatory, prebiotic). An English-language restriction was applied at the platform level in both databases; document type and publication year were not restricted at search time and were instead enforced during screening (Section 2.2). Records retrieved by all three tiers in both databases were merged into a single pooled corpus and deduplicated once using a hierarchical exact-match procedure (DOI match, then title + year match, then title + author + year match), which removed 5385 duplicate records. The full executable search strings for both databases, per-database record counts, and the deduplication procedure are provided in S1.

Reference lists of the 26 included studies and of relevant review articles identified during screening were additionally searched by hand; this step did not identify any eligible study beyond those already retrieved through the database searches (0 additional records). An English-language restriction was applied at the platform level in both databases at the point of search (S1). Title/abstract screening, full-text screening, and manual verification of the data extracted from each included study were performed independently and in parallel by two reviewers (Tran Thi Thu Suong and Hoang Ngoc Cuong); disagreements were resolved by discussion between the two reviewers until consensus was reached, with no third-party adjudicator required. This review was not prospectively registered, and no formal protocol was prepared in advance.

### 2.2. Eligibility principles

Study inclusion was guided by interpretability-oriented criteria, rather than purely formal or exhaustive selection rules, to ensure that the curated evidence could support a rigorous mechanistic evaluation of structure–activity relationships. Eligible studies were required to meet the following criteria: (i) investigate levan derived from bacterial or microbial sources; (ii) report at least one experimentally characterized structural attribute; and (iii) evaluate biological activity within antioxidant, anti-inflammatory, or prebiotic domains. Importantly, inclusion in the dataset does not imply equivalence in evidential strength or interpretive reliability. The eligibility criteria define the topographical scope of the evidence base but do not pre-determine its mechanistic value. Studies with limited structural resolution or incomplete experimental grounding were retained to reflect the broader evidentiary landscape; however, such studies were not considered equally informative for SAR interpretation. This distinction is fundamental to the present analysis, as the presence of reported associations does not inherently validate those associations as structurally or mechanistically determinable.

### 2.3. Evidence-integration tiers (post-inclusion appraisal)

Eligibility (Section 2.2) determines membership of the review corpus; it does not determine how a study may be used in synthesis. Given the heterogeneity of structural characterization and assay systems across the 26 included studies, conventional risk-of-bias tools were not considered appropriate.^46,47^ Instead, each study was assigned to one of three evidence-integration tiers based on observable, pre-defined fields (SI Table S1).

Tier A (direct within-study support) requires polymer structure, a candidate physicochemical mediator (*e.g.*, aggregation state, conformation, accessibility, rheological behaviour) and a functional outcome to be measured within the same experimental system, together with a relevant comparator/control and major structural co-variation either controlled or explicitly modelled. Tier A evidence may support a specific mediation hypothesis but does not by itself establish causality unless mediation was experimentally tested.

Tier B (indirect/triangulated support) comprises studies in which structure and function were measured but the candidate mediator was not measured, or co-variation was incompletely controlled; interpretation is limited to a constrained, non-causal association.

Tier C (contextual mapping only) comprises studies with minimal or unreliable structural characterization; these are retained to map reporting practices and heterogeneity across the corpus but are excluded from positive mechanistic inference (Table 1).

Permitted interpretive use by tier: Tier A – may support a specific mediation hypothesis; does not establish causality unless mediation is experimentally tested. Tier B – supports a constrained association or hypothesis; wording must remain non-causal. Tier C – used to map reporting and heterogeneity; excluded from positive mechanistic inference.

The criteria outlined in SI Table S1 (structural confirmation, molar-mass reliability, branching resolution, conformation evidence, assay comparability, and co-variation control) were used as the observable basis for tier assignment; the previously reported ‘context clarity’ dimension has been removed because it risked circular reasoning between structural attributes and reported outcomes.

A sensitivity analysis repeating the principal synthesis after excluding Tier C studies confirmed that the principal conclusions were retained, with the exception of the film-antioxidant observation in Section 3.8, which is explicitly identified as resting on a single lower-confidence study.

This appraisal approach is not intended to quantify study quality in a statistical sense, but to distinguish levels of interpretive validity, thereby aligning evidential classification with the conceptual objective of the review.

### 2.4. Rationale for non-meta-analytic synthesis

A quantitative meta-analysis was not undertaken, as the available evidence does not meet the conditions required for meaningful statistical aggregation.^47,62^ Structural descriptors particularly molar mass are derived from non-equivalent analytical methods and are therefore not directly comparable across studies.^29,63,64^ In addition, key structural features such as branching architecture and higher-order organization are inconsistently reported,^7,27,42,65^ while biological outcomes are generated across heterogeneous assay systems that impose distinct physicochemical constraints.^23,36^ Under these conditions, aggregation of effect sizes would risk conflating fundamentally different experimental regimes and obscuring the limitations of the underlying evidence. Accordingly, the present synthesis adopts a mechanistic interpretive approach, in which reported associations are evaluated in relation to their structural resolution and experimental context. Variability across studies is not treated as statistical noise, but as indicative of context-dependent expression, and therefore forms part of the analytical basis for examining how SAR has been constructed and, in many cases, misinterpreted within the current literature.

## 3. Evidence synthesis

### 3.1. Chemical architecture and levels of structural evidence

Bacterial levan is principally composed of β-d-fructofuranosyl residues joined through β-(2 → 6) glycosidic bonds, with β-(2 → 1)-linked fructosyl branches reported to varying extents (Scheme 1a and b). A sucrose-derived α-d-glucopyranosyl terminal residue may be present, but its abundance and analytical detectability depend on chain length and sample preparation. These linkage-level features must be distinguished from molar-mass distribution, dispersity, chain conformation, aggregation state and network formation: although frequently discussed together, they represent distinct structural levels that require different analytical evidence and cannot be inferred from one another (Scheme 1c). One- and two-dimensional NMR spectroscopy can establish linkage environments directly;^66^ methylation analysis can support quantitative linkage proportions;^67^ size-exclusion chromatography coupled with multi-angle light scattering (SEC-MALS) provides an absolute molar-mass distribution when the refractive index increment (*d*_n_/*d*_c_) and sample recovery are adequately treated; and light scattering, microscopy or rheological measurements can probe higher-order conformational and aggregation behaviour.^68^ Fourier-transform infrared (FTIR) spectroscopy alone can support functional-group assignment but cannot quantify branching density or establish a unique levan architecture.^69^ Accordingly, a reported molar mass cannot substitute for linkage structure, branching density or supramolecular state, and structure–activity claims that rely on a single descriptor without specifying which structural level was actually measured should be interpreted with this limitation in mind.

**Scheme 1:**
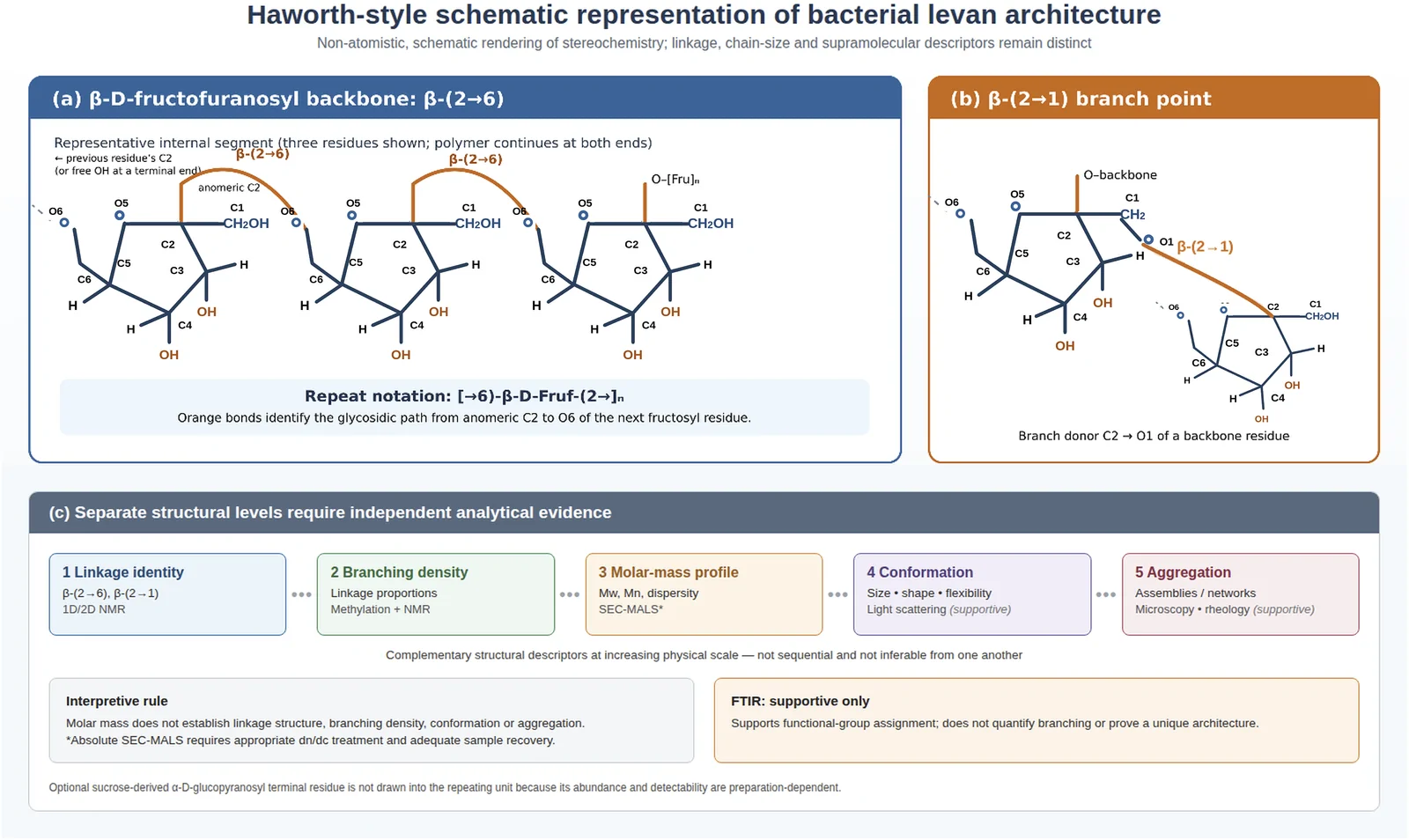
Hierarchical chemical description of bacterial levan. (a) β-(2 → 6)-linked fructofuranosyl backbone; (b) representative β-(2 → 1) branch point; (c) distinct analytical levels, from linkage identity and branching density to molar-mass distribution, conformation and aggregation, each requiring separate analytical confirmation.

### 3.2. Study selection and interpretive characteristics of the evidence base

The systematic screening process, summarized in the PRISMA 2020 flow diagram (Fig. 2), yielded 26 primary research articles from an initial pool of 18 213 records: 5385 duplicates were removed, leaving 12 828 unique records for title/abstract screening; 12 122 were excluded at that stage, leaving 706 reports sought for retrieval; 61 reports were not retrieved, 645 full texts were assessed against the eligibility criteria, and 619 were excluded with reasons recorded, yielding the 26 included studies.^40^ While this corpus provides a sufficient empirical basis for examining structure–activity relationships (SAR) in bacterial levan, its relevance lies less in its size than in the heterogeneity that limits cross-study interpretation. Across the included studies, diversity in bacterial sources is evident, with *Bacillus* spp. predominating alongside representatives of *Zymomonas*, *Acetobacter*, *Gluconobacter*, *Pseudomonas*, *Paenibacillus*, *Halomonas*, and *Enterococcus*.^13,35,37,38^ However, taxonomic variation is not the main source of analytical complexity. Rather, the key challenge arises from fragmentation in assay contexts and endpoint definitions used to represent “biological activity”.^23,36^ The evidence base encompasses fundamentally different experimental systems, including chemical reactivity assays, cell-based models, microbiota fermentation systems, and material-oriented functional platforms.^14,38,41,42^ Each of these imposes distinct physicochemical constraints such as diffusion, interfacial interaction, and enzymatic accessibility on polymer behavior.^37,52,53^ Consequently, reported antioxidant, anti-inflammatory, and prebiotic activities cannot be considered directly comparable, even when grouped under the same functional category.^13,14,35^

**Fig. 2:**
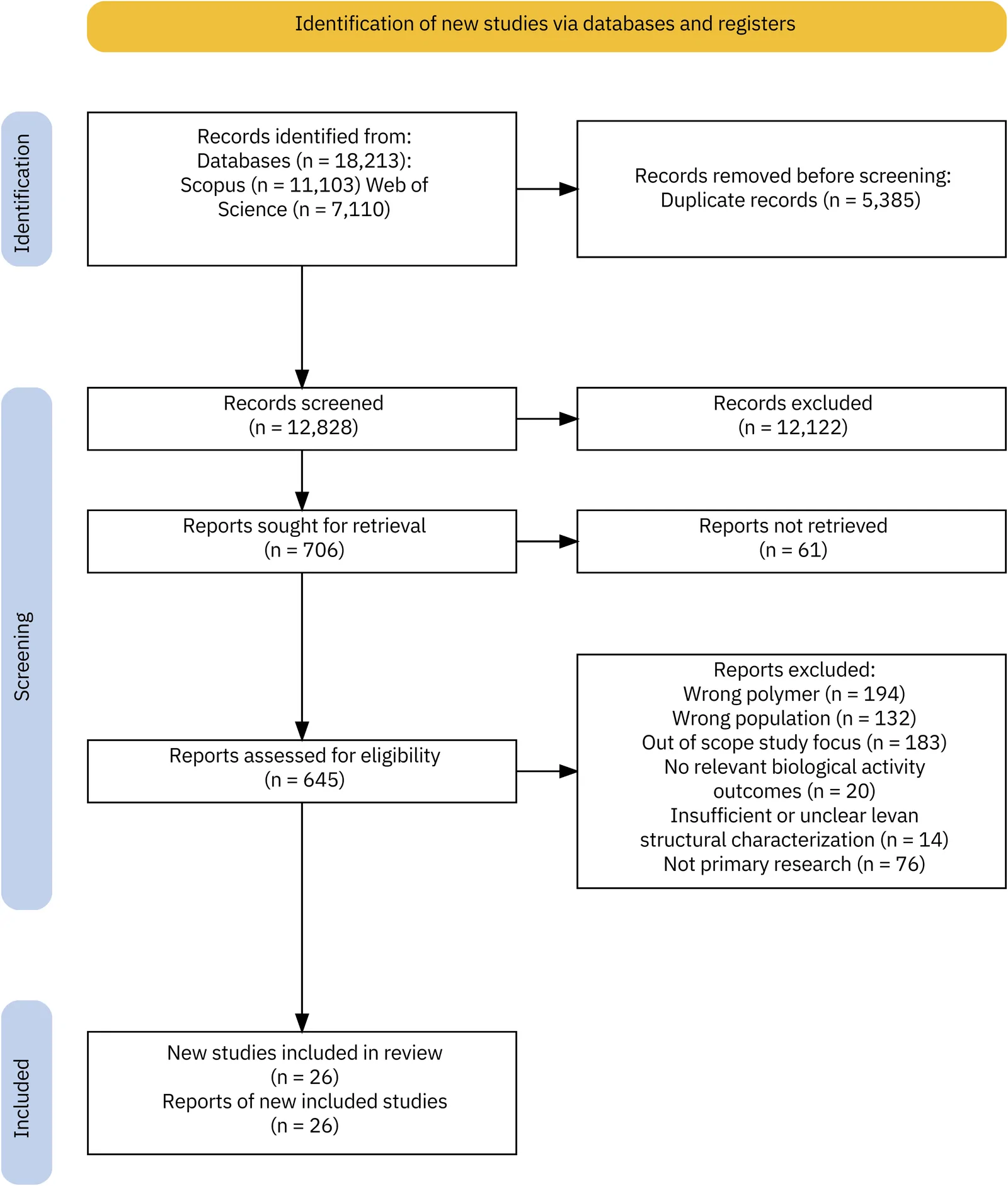
PRISMA 2020 flow diagram of study selection.

This fragmentation indicates that the diversity of reported bioactivities reflects context-dependent expression of polymer behavior rather than intrinsic multifunctionality. To avoid conflating distinct mechanistic regimes, a context-stratified analytical approach is therefore required. In this context, Table 2 is presented not as a descriptive summary, but as an analytical framework that positions each study within a dominant mechanistic regime defined by the physicochemical constraints governing functional expression.

**Table 2:** Context-stratified analytical mapping of included studies across mechanistic regimes and physicochemical constraints (*n* = 26)^a^

Study ID	Year	Producing organism	Strain/isolate	Assay context	Biological activity domain(s)	Primary endpoint type	Key outcome (one-line summary)	Mechanistic regime	Physicochemical constraint
Included_01_k_2005 (ref. 48)	2005	*Zymomonas mobilis*	ZM1	Cell-based; material matrix	Anti-inflammatory	Cytokine modulation; barrier integrity	Reduced IL-1α improved barrier function	Interface-mediated system	Surface interaction/barrier formation
Included_02_am_2012 (ref. 49)	2012	*Bacillus subtilis*	NRC1aza	Chemical; cell-based	Antioxidant	DPPH; cell viability/apoptosis	Sulfated levan derivatives showed enhanced bioactivity	Reaction-diffusion system	Radical accessibility/charge interaction
Included_03_m_2013 (ref. 50)	2013	*Bacillus licheniformis*	FRI MY-55	Microbiota/fermentation assay	Prebiotic	Bacterial growth/utilization	Low molar mass (LMW) levan promoted probiotic growth	Enzymatic accessibility system	Steric hindrance/substrate accessibility
Included_04_mat_2013 (ref. 51)	2013	*Bacillus subtilis*	Honey isolate	Chemical; cell-based	Antioxidant	ORAC; enzyme activity; apoptosis markers	Fraction dependent antioxidant and anti-initiation effects	Reaction-diffusion system	Diffusion + fraction solubility
Included_05_r_2015 (ref. 11)	2015	*Acetobacter xylinum*	NCIM 2526	Chemical; cell-based	Antioxidant; anti-inflammatory	Radical scavenging; cytokine response	Levan showed antioxidant and anti-inflammatory activity	Multi-regime system	Mixed constraints (diffusion + interaction)
Included_06_a_2019 (ref. 52)	2019	*Bacillus subtilis*	DSM 347	Microbiota/fermentation assay	Prebiotic	Growth kinetics	Levan oligosaccharides (LOS) enhanced microbial utilization	Enzymatic accessibility system	Chain length-dependent accessibility
Included_07_m_2019 (ref. 53)	2019	*Bacillus subtilis natto*	KB1	Chemical; cell-based	Antioxidant	Radical scavenging; cytotoxicity	LMW levan showed cosmetic relevant antioxidant activity	Reaction-diffusion system	Diffusion/molecular mobility
Included_08_a_2020 (ref. 65)	2020	*Bacillus subtilis*	AF17	Chemical	Antioxidant	Radical scavenging; reducing power	UHMW levans showed antioxidant activity high molar mass	Aggregation-mediated system	Aggregation/surface area effects
Included_09_f_2020 (ref. 41)	2020	*Bacillus megaterium*	PFY-147	Chemical	Antioxidant	Multi-radical scavenging	(HMW) levan showed strong antioxidant capacity	Interface-mediated system	Morphology/interfacial exposure
Included_10_tim_2020 (ref. 12)	2020	*Bacillus* sp.	Honey isolate	Organism-level functional assay; microbiota/fermentation	Anti-inflammatory; prebiotic	Tissue response; growth index	Barrier mediated gastroprotection and prebiotic effects	Barrier-mediated system	Adhesion/film formation
Included_11_sv_2021 (ref. 55)	2021	*Bacillus* sp.	4A	Microbiota/fermentation assay	Prebiotic	Growth/fermentation indices	Hydrolyzed levan improved probiotic growth	Enzymatic accessibility system	Depolymerization/accessibility
Included_12_thn_2021 (ref. 56)	2021	*Bacillus velezensis*	VTX20	Chemical	Antioxidant	Radical scavenging	Levan rich EPS showed antioxidant activity	Reaction-diffusion system	Diffusion-controlled interaction
Included_13_g_2022 (ref. 57)	2022	*Pseudomonas mandelii*	NR	Material/functional system	Antioxidant (material performance)	Film radical scavenging	Levan based films exhibited antioxidant properties	Material-mediated system	Matrix structure/diffusion limitation
Included_14_b_2023 (ref. 58)	2023	*Bacillus subtilis*	MTCC 441	Chemical	Antioxidant	Radical scavenging	HMW levan showed strong antioxidant activity	Aggregation-mediated system	Aggregation/functional group exposure
Included_15_oe_2023 (ref. 13)	2023	*Halomonas elongata*	153B	Chemical; cell-based	Antioxidant; anti-inflammatory	Radical scavenging; protein denaturation	Multifunctional antioxidant/anti-inflammatory effects	Multi-regime system	Mixed constraints
Included_16_s_2023 (ref. 59)	2023	*Bacillus* sp.	EPS003	Chemical; cell-based	Antioxidant	Radical scavenging; cytotoxicity	Antioxidant activity with low cytotoxicity	Reaction-diffusion system	Diffusion/solubility
Included_17_sa_2023 (ref. 60)	2023	*Pseudomonas aeruginosa*	HI1	Chemical; cell-based; microbiota/fermentation	Antioxidant; anti-inflammatory; prebiotic	Oxidative markers; cytokines; growth indices	Multifunctional modulation of inflammation and microbiota	Multi-regime system	System-dependent constraints
Included_18_ym_2023 (ref. 38)	2023	*Enterococcus faecium*	MC-5	Material/functional system	Antioxidant (material performance)	Film radical scavenging	LMW levan enhanced composite film bioactivity	Material-mediated system	Matrix dispersion/accessibility
Included_19_j_2024 (ref. 10)	2024	*Bacillus subtilis*	PR-C18	Chemical; microbiota/fermentation	Antioxidant; prebiotic	Radical scavenging; growth assays	LMW levan showed dual antioxidant-prebiotic effects	Hybrid system	Diffusion + enzymatic accessibility
Included_20_k_2024 (ref. 61)	2024	*Bacillus megaterium*	KM3	Chemical	Antioxidant	Radical scavenging; thermal behavior	Ordered conformation linked to antioxidant activity	Conformation-mediated system	Structural organization
Included_21_*vs.*_2024 (ref. 37)	2024	*Paenibacillus polymyxa*	88A	Chemical	Antioxidant	DPPH; ABTS scavenging	Dose dependent antioxidant activity	Reaction-diffusion system	Concentration/diffusion
Included_22_wa_2024 (ref. 14)	2024	Coculture	OP315322/OP363928	Chemical; cell-based; microbiota/fermentation	Antioxidant; anti-inflammatory; prebiotic	Radical scavenging; gene expression; growth	Enhanced multifunctional activity in coculture system	Multi-regime system	Complex interaction environment
Included_23_h_2025 (ref. 42)	2025	*Bacillus amyloliquefaciens*	JM033	Cell-based; microbiota/fermentation; organism-level functional assay	Prebiotic; immunomodulatory	Immune markers; microbiota composition	LMW levan restored immune and microbiota balance	Enzymatic + biological system	Accessibility + host interaction
Included_24_j_2025 (ref. 30)	2025	*Bacillus licheniformis*	SFLV-ZM107	Microbiota/fermentation assay	Prebiotic	Growth kinetics; fermentation indices	LMW levan enhanced probiotic fermentation	Enzymatic accessibility system	Chain length/accessibility
Included_25_nny_2025 (ref. 9)	2025	*Gluconobacter cerinus*	UELBM11	Chemical	Antioxidant	Radical scavenging	Levan showed hydroxyl and ABTS scavenging	Reaction-diffusion system	Radical interaction
Included_26_r_2025 (ref. 35)	2025	*Bacillus siamensis*	KKSB4 *et al.*	Chemical; microbiota/fermentation	Antioxidant; prebiotic	Radical scavenging; growth index	UHMW levans showed variable bioactivity	Aggregation + accessibility system	Aggregation *vs.* accessibility trade-off

a Biological activity domains in Table 2 are strictly categorized according to the review's predefined scope (antioxidant, anti-inflammatory, and prebiotic/microbiota-modulating effects). Molar mass (MW) terminology adheres to the structural stratification defined in the methods: levan oligosaccharides (LOS, <10 kDa), low (LMW, 10–100 kDa), high (HMW, 100–1000 kDa), and ultra-high (UHMW, >1000 kDa) molar masses. Abbreviations: ABTS, 2,2′-azino-bis(3-ethylbenzothiazoline-6-sulfonic acid); DPPH, 2,2-diphenyl-1-picrylhydrazyl; ORAC, oxygen radical absorbance capacity; IL, interleukin.

### 3.3. Structural diversity as a source of epistemic limitation in SAR interpretation

The structural landscape of bacterial levan reported across the included studies is characterized by pronounced heterogeneity in molar mass distribution, branching architecture, and supramolecular organization (SI Table S2).^30,42,53^ Beyond this apparent diversity, a more critical limitation emerges: structural characterization is uneven in both depth and methodological rigor, which constrains the interpretability of reported SAR patterns.

Molar mass (MW), the most frequently reported descriptor, spans several orders of magnitude from levan oligosaccharides (<10 kDa) to ultra-high molar mass polymers (>10^4^ kDa).^50,58,65^ However, the analytical approaches used to determine MW vary substantially across studies, including size-exclusion chromatography with calibration standards, viscometry-based estimation, and dynamic light scattering (DLS).^35,49,52^ These methods are not equivalent, and their outputs are not directly comparable. In particular, hydrodynamic estimates derived from DLS tend to overestimate molar mass in non-spherical polymer systems and therefore represent apparent rather than absolute values.^29^ As a result, MW frequently operates as a method-dependent proxy rather than a standardized structural parameter, rendering cross-study comparisons inherently unstable and introducing hidden variability into SAR interpretation.

In contrast, glycosidic linkage analysis consistently confirms a β-(2 → 6)-linked fructofuranosyl backbone as the defining structural feature of bacterial levan.^35,42^ However, the degree of β-(2 → 1) branching the primary source of structural variability—is quantitatively resolved in only a limited number of studies.^9,65^ In most cases, branching is described qualitatively or inferred indirectly, which precludes systematic comparison across datasets.

Similarly, higher-order structural features, including conformation and supramolecular organization, are predominantly inferred from proxy-based methods (*e.g.*, Congo red assays, SEM morphology, rheological behavior) rather than direct structural measurements.^37,38,41^ These descriptors therefore carry substantial uncertainty and cannot be treated as robust variables within SAR analysis.

Taken together, these observations indicate that the structural basis of current SAR evidence is not only heterogeneous but also partially under-resolved. Consequently, the reliability of structure-dependent interpretations is constrained less by data availability than by the uneven quality and comparability of structural characterization.

In this context, SI Table S2 is not presented as a descriptive summary, but as a structural interpretability map that delineates the conditions under which reported structural descriptors can support SAR inference. Molar mass values are therefore stratified according to methodological reliability, and studies lacking sufficient structural resolution are restricted to descriptive or context-dependent interpretation.

### 3.4. Chemical antioxidant assays

Chemical antioxidant assays constitute the largest single evidence regime in this review, comprising nineteen of the twenty-six included studies (Table 1), predominantly at the Tier B (indirect/triangulated) level,^65^ with three Tier C studies.^49,57,61^ Across this regime, higher apparent radical-scavenging activity is frequently reported for higher-molar-mass fractions;^41,49,65^ however, attributing this pattern to increased intrinsic redox reactivity of the polymer backbone is not supported by the available mechanistic evidence.^37,41,56^ Instead, the pattern is more consistently explained by aggregation-driven interfacial phenomena, including increased effective surface area, altered microenvironmental stabilization of reactive intermediates, and enhanced functional-group presentation at phase boundaries.^35,58,65^ These effects are highly sensitive to assay-specific conditions – solvent system, radical species and reaction kinetics – which are rarely harmonized across studies.^37,70^

Complementary evidence links particle-level morphology to antioxidant response: porous or rough surface morphologies are associated with enhanced antioxidant activity in chemical assays, plausibly reflecting increased interfacial exposure rather than a fundamental change in chemical structure.^9,10,37,59^ The only chemically modified sample in the corpus – a sulfated levan derivative^49^ – showed increased antioxidant activity relative to the native fraction; because sulfation co-occurs with changes in degree of substitution, molar-mass distribution and solubility that were not independently resolved, this remains an exploratory, single-study observation rather than evidence of a specific structure–activity relationship.^37,49^ Across the remaining antioxidant-regime studies, reported outcomes are qualitatively consistent with this interfacial/aggregation-mediated pattern – for example fraction-dependent effects,^51^ dose-dependent responses^37^ and radical-species-specific scavenging^9^ – but structural and assay heterogeneity precludes a single quantitative structure–activity rule for this regime. A genuine covariation control isolating molar mass or aggregation state from co-varying structural features was reported in only one study; no other study in this regime held branching, dispersity or assay conditions constant while varying a single structural parameter, which further constrains the causal strength of the associations described above.

### 3.5. Cell-based inflammatory responses

Cell-based inflammatory evidence is available for six studies (Table 1), of which only one – an interface-mediated barrier assay reporting reduced IL-1a and improved epithelial barrier function^48^ – was evaluated as a single-regime (Tier B) inflammatory endpoint. The remaining five studies report anti-inflammatory activity jointly with antioxidant, prebiotic and/or gastrointestinal endpoints within the same experimental system.^11–14,60^ Among these five, one reports a partially controlled mechanistic pathway (NF-κB/COX-2) linking a barrier/adhesion-forming physicochemical state to the inflammatory readout; the remaining four report no comparator isolating a physicochemical variable from the inflammatory outcome. Because structure, physicochemical state and the inflammatory endpoint were rarely isolated from co-measured outcomes, cell-based inflammatory evidence in this corpus is best read as compatible with, rather than independently demonstrating, the interfacial-mediation model proposed for the antioxidant regime; it is discussed further as part of the multi-regime pattern in Section 3.9.

### 3.6. Microbial fermentation and prebiotic systems

Nine studies assessed microbial fermentation or prebiotic outcomes (Table 1). The most consistent directional trend in the entire evidence base is observed here: lower-molar-mass fractions and levan oligosaccharides are repeatedly associated with enhanced microbial utilization and probiotic growth,^12,30,55^ including the review's Tier A study in this regime.^30^

This pattern is more plausibly explained by transport-mediated accessibility than by an intrinsic functional superiority of low-molar-mass material: reduced chain length lowers steric hindrance and improves enzymatic access, thereby facilitating substrate uptake.^30,35^

Rheological behaviour and chain entanglement further modulate this relationship – more compact or entangled structures reduce fermentation efficiency, plausibly through restricted substrate accessibility, whereas more dispersed configurations facilitate microbial utilization.^30,35,52^ One study reported variable bioactivity for an ultra-high-molar-mass levan, illustrating that the accessibility-driven pattern does not extrapolate linearly to the upper end of the molar-mass range.^35^ This regime contains the review's only genuine within-system covariation control: a direct comparison of low- and high-molar-mass fractions from the same preparation, with molar mass confirmed by absolute GPC-MALLS. One of the multi-regime studies discussed below additionally offers a partially controlled mechanistic pathway (NF-κB/COX-2) linking barrier formation to its gastrointestinal/prebiotic outcome; no other study in this regime reports a comparator isolating a single structural or physicochemical variable. As in the inflammatory regime, several of these studies also report antioxidant, anti-inflammatory or gastrointestinal outcomes within the same system,^10,12–14,60^ and are treated jointly with those endpoints in Section 3.9.

### 3.7. Gastrointestinal-protection models

Only two studies in the corpus assessed a gastrointestinal-protection endpoint (Table 1): a barrier-mediated gastroprotection and prebiotic assay (Tier B),^12^ and a multifunctional study reporting joint modulation of inflammation and microbiota (Tier B).^60^ Both report the gastrointestinal outcome alongside at least one other regime rather than in isolation. Only the first offers a partially controlled mechanistic pathway (NF-κB/COX-2) linking a barrier/adhesion-forming physicochemical state to its protective outcome; neither study reports a full covariation control isolating a single structural variable. Given this evidence base of two studies, neither independently powered to isolate a gastrointestinal-specific structure–function relationship, gastrointestinal-protection findings for bacterial levan should currently be treated as a preliminary, hypothesis-generating observation rather than a validated regime-specific pattern.

### 3.8. Material and film performance

Three studies evaluated levan-based material or film performance (Table 1). Antioxidant activity has been reported for a levan-incorporated film in a single Tier C study, attributed to matrix structure and diffusion limitation within the film rather than to the polymer's intrinsic reactivity,^57^ consistent with the aggregation/interfacial-exposure interpretation proposed for the chemical antioxidant regime (Section 3.4). The remaining two material-performance studies report film- or matrix-relevant outcomes jointly with antioxidant and/or anti-inflammatory endpoints in the same system,^13^ or within a hybrid diffusion/enzymatic-accessibility system combining antioxidant and prebiotic readouts.^10^ As with the cell-based and fermentation regimes, the small number of single-purpose material studies and the recurrence of multi-regime designs limit generalization; material-performance evidence is therefore best interpreted as supportive of, rather than independent from, the physicochemical-mediation model developed across the other regimes. None of the three studies in this regime reports a covariation control isolating molar mass, dispersity or matrix structure from the other co-varying features of the film system.

### 3.9. Cross-regime interpretation

Two structural features recur across the regime-specific evidence in Sections 3.4-3.8 without mapping cleanly onto any single regime, and are treated here as cross-regime, hypothesis-level observations rather than assigned to a single assay context.

#### 3.9.1. Molar mass across regimes

The prevailing interpretation of molar mass (MW) as a determinant of bioactivity in bacterial levan is conceptually misleading.^49,51^ As shown in Table 3, MW exhibits domain-dependent rather than uniform associations with functional outcomes:^35,37,53^ an inverse MW-activity relationship in fermentation/prebiotic systems (Section 3.6) coexists with a positive apparent association between higher MW and antioxidant activity (Section 3.4) – a divergence consistent with regime-specific physicochemical mediation (accessibility *versus* aggregation) rather than with a single causal role for MW. The intermediate MW range (10–100 kDa) further illustrates the limits of descriptor-based interpretation, showing inconsistent and sometimes contradictory outcomes across domains;^49,51^ this is best explained by competing, regime-dependent contributions of diffusion, aggregation and accessibility rather than by anomalous behaviour.^30,35,52^ Taken together, MW should not be interpreted as a causal determinant of bioactivity, but as a method- and regime-dependent descriptor whose apparent effects arise from underlying physicochemical processes that differ by assay context.

**Table 3:** Context-constrained SAR evidence linking molar mass of bacterial levan to biological activities through physicochemical mediation

Molar mass category (kDa)	Biological activity domain	Predominant directional^a^	Qualitative evidence consistency^b^	Studies contributing to this category	Contextual interpretation and limitations	Physicochemical mediator	Interpretation validity
<10	Prebiotic/microbiota-modulating	↑	Moderate-strong	Included_06 (LOS ∼2.3), included_24 (LML, ∼7.7)	Consistent positive prebiotic effects were discussed by the study authors in terms of improved enzymatic accessibility of low-DP levans/oligosaccharides	Enzymatic accessibility/reduced steric hindrance	Mechanistically supported
10–100	Prebiotic/microbiota-modulating	↑	Moderate	Included_10 (∼21), included_19 (∼34), included_22 (∼11.5), included_23 (∼17.6)	Generally supportive of microbial growth across models, though magnitude varies by strain and assay context	Enzymatic accessibility + diffusion	Context-dependent
10–100	Prebiotic/microbiota-modulating	↓	Limited (within-study comparisons only)	Included_06: native levan (∼29.8 kDa); included_24: HML (∼25.8 kDa)	Higher-MW levan supported less bacterial growth or fermentation than the lower-MW samples. HML in included_24 showed greater thermal and structural stability	Reduced enzymatic accessibility at higher MW	Supported by within-study comparisons
10–100	Antioxidant	↑/↔	Mixed	Included_04 (9.5–21.8), included_22 (∼11.5), included_07 (∼37.6), included_18 (∼24), included_19 (∼34)	Direction varies by assay type (*e.g.*, DPPH *vs.* ORAC) and fraction specific physicochemical properties	Diffusion + solubility + fraction heterogeneity	Context-dependent
100–1000	Antioxidant	↑	Limited	Included_25 (∼878)	Positive association reported in a small number of studies; effects often discussed in relation to morphology or compositional features rather than molecular size alone	Interfacial exposure/morphology	Context-dependent
>1000	Antioxidant	↑	Moderate	Included_08 (20 000 kDa), included_09 (∼1878), included_14 (∼2831), included_15 (∼3260), included_21 (∼1900)	Activity often interpreted in relation to aggregation state, surface exposure, or functional group abundance rather than molecular size alone	Aggregation/surface interaction	Context-dependent
>1000	Prebiotic/microbiota-modulating	↓/↔	Limited (single study)	Included_26 (UHMW; DLS-apparent)	Reduced effects were discussed by the study authors in terms of limited enzymatic accessibility at higher molecular size; strain-specific or method-dependent exceptions reported	Steric hindrance/diffusion limitation	Mechanistically supported
Variable MW (fractionated systems)	Anti-inflammatory	Context dependent	Weak	Included_10, included_17, included_22	Outcomes depend strongly on biological model and proposed mechanism (*e.g.*, adhesion, immunomodulation) rather than MW alone	Interface interaction/biological modulation	Context-dependent
>1000	Anti-inflammatory	↑	Limited (single study)	Included_01 (∼2250)	Reduced IL-1α and improved epithelial barrier integrity were reported in a single high-MW self-assembling system; the association is discussed by the study authors in relation to interfacial/barrier-modulating structure rather than molecular size alone	Interfacial interaction/self-assembly	Context-dependent

a Directional associations reflect the most frequently reported qualitative trends: (↑) positive correlation/enhancement; (↓) negative correlation/attenuation; (↔) no consistent association observed.

b Evidence consistency is graded as: strong (≥3 concordant studies); moderate (2–3 studies with minor discrepancies); mixed (conflicting directions); weak/limited (single study or highly context specific outcomes). MW values are weight average molar masses (kDa) as assigned to predefined polymer scale categories.

Branching architecture is often considered the primary structural variable beyond molar mass in bacterial levan.^11,42,65^ However, its role in structure–activity relationships remains insufficiently defined across all regimes, largely due to the limited availability of quantitative and comparable structural data: the β-(2 → 6) backbone is consistently conserved, whereas the degree of β-(2 → 1) branching is quantitatively resolved in only a small subset of studies employing methylation analysis or advanced NMR;^9,42,65^ in most cases it is described qualitatively or inferred from spectral similarity, without a standardized branching index.^37,42,65^ Branching also does not vary independently of molar mass, dispersity and higher-order conformation, making it difficult to isolate its specific contribution to any single regime's outcome.^37,42^ Increased activity has been reported in more highly branched fractions in some studies, but in the absence of controlled structural variation this is more appropriately attributed to co-varying structural features than to branching alone;^42,53^ branching is therefore best treated as a plausible cross-regime modulator rather than a regime-specific determinant.

#### 3.9.2. Multi-regime studies and the two unclassified studies

Five studies report outcomes across two or more regimes within the same experimental system,^11,13,14,60^ most commonly combining antioxidant, anti-inflammatory, prebiotic and/or gastrointestinal endpoints.^10^ These studies are counted in each relevant regime section above and are not treated as providing regime-independent confirmation, since the co-measured endpoints share the same structural preparation and are not statistically or experimentally independent. In addition, two studies could not be assigned to a single predefined regime from the extracted data:^42,50^ one reports enzymatic-accessibility-mediated microbial utilization consistent with the fermentation/prebiotic pattern above, and one reports combined immune and microbiota-related outcomes.

#### 3.9.3. Consistency and certainty of SAR evidence across regimes

The overall SAR evidence for bacterial levan reveals a pattern of regime specific consistency alongside pronounced cross-regime heterogeneity. Among the examined regimes, the most consistent directional trend is observed in fermentation/prebiotic systems, where lower-MW fractions are repeatedly associated with enhanced microbial utilization across multiple studies;^30,52,55^ however, this relationship is more appropriately interpreted in terms of accessibility and transport-related properties than as evidence of intrinsic structural superiority.

In contrast, antioxidant and inflammatory regimes demonstrate considerably lower consistency, with reported effects varying across assay types and experimental conditions,^23,35,37^ plausibly reflecting underlying factors that are often not fully characterized, including branching degree, dispersity, aggregation state and compositional heterogeneity.^41,42,48^ Three limitations constrain the interpretability of current SAR evidence across all regimes: incomplete structural characterization, particularly of branching architecture and higher-order organization;^41,53^ substantial heterogeneity across experimental systems that limits comparability and complicates cross-study synthesis;^14,23,60^ and co-variation among structural parameters that makes it difficult to isolate the contribution of individual descriptors as independent determinants of function. In light of these considerations, current SAR conclusions are best understood as qualitative and regime-conditioned rather than quantitative or predictive; the observed patterns are more appropriately interpreted as emergent outcomes arising from the interplay between polymer structure, physicochemical behavior and experimental regime.

## 4. Discussion

The review supports one robust negative conclusion and a more limited positive proposition. First, the current literature does not justify treating molar mass as a universal causal determinant of bacterial levan function. Second, diffusion, aggregation, accessibility and interfacial interaction provide chemically plausible candidate mediators, but the included studies rarely measured these variables together with structure and function. The proposed framework should therefore be read as a falsifiable model for experimental design, not as a demonstrated mediation pathway. Its value lies in specifying what must be measured within the same system and which cross-study comparisons are currently inadmissible.

### 4.1. Reframing SAR: from descriptor-based interpretation to mechanistic constraint

The apparent inconsistency of reported structure–activity relationships (SAR) in bacterial levan does not arise solely from biological variability, but reflects a pronounced asymmetry in evidential strength across the literature. As indicated by the evidence appraisal matrix (Table 1), mechanistically interpretable signals are confined to a limited subset of structurally well-resolved studies, whereas a substantial proportion of the evidence base is characterized by incomplete structural characterization and heterogeneous experimental contexts.

This imbalance provides a direct basis for understanding the variability of SAR patterns reported across studies. Rather than indicating contradictory structure–function relationships, the observed inconsistency emerges from differences in structural resolution, assay comparability, and context alignment, which collectively constrain the interpretability of reported associations. In this sense, SAR variability should be interpreted not as intrinsic unpredictability, but as a consequence of uneven evidential foundations within the current literature.

Within this evidential landscape, SAR in bacterial levan has commonly been approached through a descriptor-based perspective, in which individual structural parameters most notably MW are treated as primary determinants of biological activity.^41,49,71^ However, the present synthesis indicates that such interpretations are not supported by the underlying structure of the available evidence. Structural attributes are not consistently defined, nor are they independently controlled across studies, thereby limiting their capacity to support causal inference.^29,33,72^

Instead, these descriptors are more appropriately understood as proxies for underlying physicochemical constraints, including diffusion, aggregation, and molecular accessibility, which govern how the polymer is expressed within a given experimental system.^2,24,32^ Under these conditions, reported associations between structure and activity cannot be interpreted independently of the physicochemical and contextual parameters within which they are observed.

Importantly, the presence of biological activity does not, in itself, constitute evidence of a structure–function relationship. Mechanistically interpretable inference requires sufficient structural resolution in conjunction with methodological transparency and context alignment.^28,40,47^ As indicated in Table 1, only a subset of studies meets these conditions, whereas the majority of reported findings remain context-dependent and therefore cannot be attributed to structural features alone.

Accordingly, SAR in bacterial levan is more appropriately understood as a constrained and context-dependent phenomenon, in which structural attributes influence functional outcomes indirectly through their effects on physicochemical behavior under specific experimental conditions.

### 4.2. Molar mass: a proxy for accessibility rather than a causal variable

Molar mass is the most frequently reported structural parameter in the levan literature and is often interpreted as a primary determinant of biological activity.^2,8^ The present analysis suggests that this interpretation is conceptually limited.

In microbiota-related systems, lower-MW fractions are consistently associated with enhanced microbial utilization.^30,32^ However, this pattern is more plausibly explained by increased enzymatic accessibility and reduced steric hindrance, rather than by intrinsic structural superiority.^33,73^ In contrast, higher-MW fractions frequently show increased activity in chemical antioxidant assays,^41,65^ which is more consistent with aggregation-related interfacial effects than with inherent redox properties of the polymer backbone.^1,23^

These domain-specific trends do not converge into a unified SAR pattern. Instead, they reflect different physicochemical mechanisms operating under distinct assay constraints. Under these conditions, MW functions as a context-sensitive proxy for transport, accessibility, and aggregation behavior, rather than as a causal variable.

In addition, the interpretability of MW is constrained by methodological variability.^63,64^ As shown in SI Table S2 and reflected in Table 1, MW values are derived from non-equivalent analytical approaches, including relative calibration methods and hydrodynamic estimates, which are not directly comparable.^29,74^ This further limits the robustness of MW-based SAR interpretation.

Taken together, the widespread reliance on MW as an explanatory variable represents a reduction of inherently multi-scale polymer behavior to a single descriptor, which does not adequately capture the mechanisms underlying functional outcomes.

### 4.3. Branching architecture: plausible relevance under limited structural resolution

Branching architecture is frequently proposed as a secondary determinant of levan bioactivity. However, its role within SAR remains insufficiently resolved due to limited availability of quantitative and comparable structural data.^29,33^

Although β-(2 → 1) branching is recognized as a key source of structural variability beyond the β-(2 → 6) backbone, it is only quantitatively characterized in a small number of studies.^28,75^ In most cases, branching is described qualitatively or inferred indirectly, which limits the possibility of systematic comparison across studies.

Moreover, branching does not vary independently from other structural parameters.^5,72^ Changes in branching are often accompanied by variations in molar mass, dispersity, and supramolecular organization, making it difficult to isolate its specific contribution to biological activity.^2,29^ Under these conditions, reported associations between branching and function cannot be interpreted as mechanistically grounded.

While some studies suggest enhanced activity in more branched systems,^9,56^ these observations are not based on controlled structural variation and therefore remain interpretively limited. Branching may plausibly influence physicochemical properties such as hydration, packing, and accessibility, but its role as an independent determinant of activity is not established.

Accordingly, current SAR claims related to branching should be regarded as structurally under-resolved and not sufficient to support causal inference.

### 4.4. Conformation and aggregation: mediators of functional expression

Higher-order structural features, including conformation and aggregation state, are increasingly invoked to explain variations in levan bioactivity.^7,29^ The present analysis suggests that these features are more appropriately interpreted as mediators of functional expression rather than independent descriptors.

Most studies rely on indirect proxies such as Congo red assays, SEM/AFM observations, or rheological measurements to infer conformational organization.^6,9^ These approaches do not provide standardized or directly comparable measures of polymer conformation, and therefore introduce uncertainty into SAR interpretation.^28,75^

Despite these limitations, a consistent pattern can be identified. Functional outcomes are closely associated with properties that influence interfacial exposure, diffusional transport, and substrate accessibility.^1,76^ For example, increased antioxidant activity observed in certain systems is more plausibly linked to aggregation-mediated surface effects than to intrinsic chemical reactivity.^41,65^ Similarly, reduced microbial utilization in highly entangled or viscous systems reflects limitations in accessibility rather than absence of functional potential.^32,35^

These observations support a model in which conformation and aggregation act as intermediate states through which structural attributes are translated into context-dependent functional outcomes. However, because these features are rarely measured directly and often co-vary with other structural parameters, their role cannot be isolated within conventional SAR frameworks.

### 4.5. Evidence asymmetry and limits of mechanistic inference

A key observation emerging from the evidence appraisal matrix (Table 1) is the asymmetry between the number of reported associations and the strength of mechanistically interpretable evidence.

Although many studies report positive relationships between levan structure and biological activity, only a limited subset provides sufficient structural resolution and contextual alignment to support mechanistic interpretation.^28,77^ A substantial proportion of the literature remains exploratory, with findings that are dependent on specific assay conditions and cannot be generalized.^36,56,78^

This asymmetry suggests that the apparent consistency of reported bioactivities particularly in antioxidant and prebiotic domains does not reflect convergence toward a unified structure–function relationship. Rather, it reflects repeated observations of context-dependent outcomes under heterogeneous experimental conditions.

Under these conditions, variability across studies should not be treated as noise to be resolved through aggregation, but as an intrinsic feature of polymer-based systems.^27,79^ Mechanistic inference must therefore be restricted to cases where structural and experimental conditions are sufficiently aligned.

This asymmetry does not merely reflect data heterogeneity, but indicates a structural limitation in how SAR has been constructed and interpreted within the current literature.

### 4.6. Translation of levan bioactivity across digestion, bioavailability, and food systems

#### 4.6.1. Gastrointestinal digestion and structural accessibility of levan

Beyond assay-dependent behavior, the functional expression of bacterial levan must be interpreted within the context of gastrointestinal processing, where physicochemical constraints are further modulated by digestion dynamics.^24,39,80,81^ Unlike digestible carbohydrates, levan is resistant to enzymatic hydrolysis in the upper gastrointestinal tract due to the absence of specific human enzymes capable of cleaving β-(2 → 6) fructosyl linkages.^2,39^ As a result, the polymer largely reaches the colon in an intact or partially modified form, where its accessibility to microbial metabolism becomes the dominant determinant of functional expression.^82,83^

Consistent with the accessibility-based role of molar mass established above (Section 4.2), lower molar mass fractions and oligosaccharides exhibit enhanced fermentability due to reduced steric hindrance and increased surface exposure, facilitating microbial uptake and enzymatic degradation.^30,33,73^ In contrast, high molar mass or highly entangled structures may limit accessibility, delaying or reducing fermentation efficiency.^32,35^

Importantly, these digestion-mediated transformations indicate that SAR observed under *in vitro* conditions cannot be directly extrapolated to *in vivo* nutritional functionality. Instead, structural attributes define a range of possible physicochemical states, which are further reshaped during gastrointestinal transit. Consequently, the effective structure interacting with gut microbiota may differ substantially from the initially characterized polymer, introducing an additional layer of context dependency into SAR interpretation.

#### 4.6.2. Bioavailability and functional expression beyond absorption paradigms

In contrast to small bioactive molecules, the biological effects of levan are not primarily mediated through systemic absorption, but through localized interactions within the gastrointestinal environment.^3,84^ As a high molar mass polysaccharide, levan exhibits minimal direct bioavailability in the classical pharmacokinetic sense.^2,85^ Instead, its functional relevance arises from indirect mechanisms, including modulation of gut microbiota, interaction with intestinal barriers, and physicochemical effects within the luminal environment.^15,86^

This distinction has important implications for interpreting antioxidant and anti-inflammatory activities. Chemical antioxidant assays, which are frequently used to characterize levan bioactivity, measure radical scavenging capacity under simplified conditions that do not reflect the spatial and kinetic constraints of the gastrointestinal tract.^23,24,87^*In vivo*, antioxidant effects are more likely to arise from indirect pathways, including modulation of microbial metabolism and attenuation of oxidative stress *via* microbiota-derived metabolites.^4,19^

Similarly, anti-inflammatory effects observed in cell-based systems may not directly translate to systemic outcomes but instead reflect localized interactions at the gut interface.^42,88^ For instance, film-forming or barrier-associated properties of levan may contribute to epithelial protection and modulation of inflammatory signaling, rather than acting through direct receptor-mediated mechanisms.^12,60^

Taken together, these observations indicate that bioavailability in the context of levan should be interpreted in functional rather than absorptive terms, emphasizing interaction within the gastrointestinal environment rather than systemic distribution.

#### 4.6.3. Gut microbiota interactions and mechanism-level interpretation

The most consistent and mechanistically plausible functional domain for levan is its interaction with gut microbiota. However, even within this domain, the relationship between structure and function is not direct, but mediated by accessibility, enzymatic specificity, and microbial community composition.^4,84^

Low molar mass levans and oligosaccharides are more readily utilized by microbial populations due to their increased accessibility, leading to enhanced fermentation and production of short-chain fatty acids (SCFAs).^30,42,52^ These metabolites contribute to downstream physiological effects, including modulation of immune responses and maintenance of gut barrier integrity.^15,18,89^

In contrast, higher molar mass fractions may exhibit delayed or selective fermentation, potentially supporting distinct microbial niches within the gut ecosystem.^32,90,91^ This suggests that different structural forms of levan do not exhibit uniform functionality but rather contribute to a spectrum of microbiota-mediated outcomes.

Importantly, microbial utilization is influenced not only by polymer size, but also by conformation, aggregation state, and interactions with other dietary components.^35,92^ These factors collectively determine enzymatic accessibility and metabolic efficiency, reinforcing the view that prebiotic function emerges from multi-level interactions rather than direct structural effects.

#### 4.6.4. Food matrix interactions and processing-dependent transformations

In real food systems, levan is embedded within complex matrices where its structure and functionality are influenced by processing conditions and interactions with other components.^93–95^ These factors introduce additional variability into SAR interpretation that is rarely accounted for in experimental studies.

Processing conditions including thermal treatment, pH variation, and shear forces can induce structural modifications such as depolymerization, aggregation, or conformational rearrangement.^29,96^ Such changes may alter molar mass distribution and supramolecular organization, thereby influencing physicochemical behavior and functional expression.^26,52^

Furthermore, interactions with proteins, lipids, and other polysaccharides can modulate dispersion state, viscosity, and water-binding capacity, which in turn affect biological accessibility during digestion.^25,34^ For example, increased viscosity or gel formation may restrict diffusion and enzymatic exposure, thereby reducing microbial utilization despite favorable intrinsic structural properties.^24,31^

From a technological perspective, these same properties contribute to food texture, stability, and functionality.^54,97,98^ However, their influence on digestion and bioactivity highlights the need to consider food processing and matrix interactions as integral components of structure–function relationships, rather than as secondary variables.

#### 4.6.5. Integration across structural, physicochemical, and nutritional levels

The incorporation of digestion, bioavailability, microbiota interaction, and food processing into SAR interpretation reveals that levan bioactivity emerges from a multi-level system rather than a direct structure–function relationship.

This multi-level interaction can be conceptually represented as a constrained, modifier-based framework in which food-matrix, processing and digestion context act on physicochemical behavior and biological-interface expression rather than forming a strict sequential progression (Fig. 1).

Building on this interpretive framework, functional outcomes arise from the interaction between polymer properties (including molar mass and branching) and the physicochemical and biological environments in which they are expressed, rather than from these descriptors alone.

From a practical food system perspective, these findings indicate that the functional performance of levan cannot be optimized through structural descriptors alone, but must be considered in relation to the intended application environment.

For formulation purposes, lower molar mass fractions may be more suitable for applications targeting microbiota modulation, where enzymatic accessibility and fermentability are critical.^30,32,52^ In contrast, higher molar mass or aggregated forms may be more appropriate in systems where interfacial properties, film formation, or viscosity contribute to functional performance, such as in food structuring or protective matrices.^34,35,54^

Importantly, these functional advantages are context-dependent and may not be transferable across applications. A structural configuration that enhances performance in a chemical assay or material system may not yield equivalent benefits in gastrointestinal or microbiota-mediated contexts.

Accordingly, effective utilization of levan in food systems requires alignment between structural properties, physicochemical behavior, and the specific constraints of the target application, including processing conditions, matrix composition, and digestion dynamics.

### 4.7. Implications for experimental design and interpretation

The limitations identified in the current evidence base point to several directions for improving both experimental design and interpretive practice.

First, structural characterization should extend beyond single-parameter reporting to include multi-dimensional descriptions of polymer architecture, including molar mass distribution, branching, and higher-order organization.^28,33^ Standardized and comparable measurement approaches are essential.^75,77^

Second, experimental systems should explicitly consider physicochemical mediation. Observed functional outcomes should not be interpreted as direct expressions of intrinsic activity, but as the result of interactions between polymer properties and assay conditions.

Third, structural parameters should be varied independently where possible. The co-variation of molar mass, branching, and conformation in existing studies limits the ability to establish causal relationships.^2,29^

Finally, functional outcomes should be interpreted within the context of the specific experimental system in which they are measured.^23,24^ Direct comparison across fundamentally different assay types introduces conceptual inconsistency and obscures underlying mechanisms.

### 4.8. Toward a constrained, context-aware SAR framework

Based on the present synthesis, SAR in bacterial levan can be reformulated within a constrained, multi-level framework.

At the structural level, polymer architecture defines a range of physicochemical possibilities.^33,42^ At the physicochemical level, properties such as diffusion, aggregation, and accessibility determine how the polymer interacts with its environment.^29,32^ At the biological-interface level, these physicochemical properties are translated into a measurable outcome through a specific system – a chemical assay, a cellular or tissue system, or the gut microbiota – with the surrounding experimental context (assay type, solvent, reaction kinetics) acting as an effect modifier rather than a discrete level in its own right.^23,60^

Within this framework, functional effects are not directly determined by structural descriptors, but emerge from interactions across these levels. Structural attributes influence the range of possible behaviors, but do not uniquely determine functional outcomes.

This framework does not aim to establish predictive rules, but to provide a more coherent basis for interpreting SAR evidence in polymer-based systems.

### 4.9. What constitutes interpretable SAR evidence in levan research

The present analysis indicates that not all reported structure–activity relationships are equally interpretable. For SAR claims in bacterial levan to support mechanistic inference, several conditions must be met.

Structural attributes must be quantitatively and reliably characterized using methods that enable comparison across studies.^75,77^ These attributes should be varied independently, or their co-variation explicitly addressed.^29,33^ The experimental context must be clearly defined, allowing a plausible mechanistic link between structure and observed function.^24,99–101^ Finally, physicochemical mediation should be explicitly considered.^2,32^

In the absence of these conditions, reported associations should be interpreted as context-dependent observations rather than as evidence of intrinsic structure–function relationships.

This distinction defines the boundary between descriptive reporting and mechanistic understanding, and is therefore central to advancing SAR analysis in bacterial levan research.

## 5. Conclusions

Available evidence does not support a universal descriptor-driven SAR for bacterial levan. In particular, molar mass is not independently interpretable when branching, dispersity, conformation, aggregation, purity and assay conditions co-vary. The evidence is strongest when conclusions are restricted to a single experimental regime and to studies with adequate structural characterization and controls. The proposed structure-physicochemical state-function pathway remains a testable model because direct within-study mediation evidence is scarce.

Future work should combine linkage and branching analysis, absolute molar-mass characterization, direct measurement of aggregation or accessibility, and matched functional assays under controlled comparisons. Such studies would allow the field to move from post hoc association toward reproducible polymer structure–function chemistry.

Future research should prioritize integrated experimental designs that combine high-resolution structural characterization with context-relevant functional evaluation, thereby enabling the generation of evidence that is both reproducible and mechanistically interpretable within realistic application settings.^75,77^

From a food science perspective, these findings underscore the need to move beyond descriptor-based selection toward context-aware design. Structural parameters alone are insufficient to anticipate functionality unless considered in relation to processing conditions, matrix interactions, and gastrointestinal transformation.^39,93^

Accordingly, advancing the application of levan in food systems requires the integration of structural, physicochemical, and contextual constraints within experimental design. Such an approach is essential for bridging the gap between laboratory-scale bioactivity and real-world nutritional functionality, and for enabling more reliable, application-relevant deployment of levan in functional foods and nutraceutical systems.

## Author contributions

Tran Thi Thu Suong: conceptualization, methodology, data curation, formal analysis, investigation, writing – original draft. Nguyen Phung Kieu: formal analysis, validation, writing – review & editing. Hoang Ngoc Cuong: investigation, formal analysis, validation, data curation. Tran Dinh Thang: formal analysis, validation, writing – review & editing. Nguyen Ngoc Tuan: conceptualization, resources, supervision, project administration, writing – review & editing. All authors have read and approved the final version of the manuscript.

## Conflicts of interest

There are no conflicts to declare.

## Supplementary Material

RA-016-D6RA04924A-s001

RA-016-D6RA04924A-s002

## Data Availability

No new experimental data were generated for this review. The systematic search, screening, evidence-tier appraisal, and study-level extraction data underlying this review are provided as supplementary information (SI). Raw database export files (Scopus and Web of Science) are archived by the corresponding author and available upon reasonable request. Supplementary information: the systematic review supporting data, including S1: search history summary; S2: full-text exclusion list with reasons (*n* = 619); S3: PRISMA 2020 accounting; and S4: study-level extraction dataset for the 26 included studies. See DOI: https://doi.org/10.1039/d6ra04924a.
