# Supplementary material for: A systematic review of structure–activity relationships in bacterial levan: physicochemical mediation and context-dependent functionality
Source: RSC Adv. 2026 Oct 5;16(46):59022–44. doi: 10.1039/d6ra04924a (PMC13636291; doi:10.1039/d6ra04924a)
Supplement: Supplementary file 1 [file RA-016-D6RA04924A-s001.pdf]

Supplementary Information (SI) for RSC Advances.
This journal is © The Royal Society of Chemistry

PRISMA 2020 Checklist

Section and Topic	Item #	Checklist item	Location where item is reported
**TITLE**			
Title	1	A systematic review of structure–activity relationships in bacterial levan: physicochemical mediation and context-dependent functionality	Title
**ABSTRACT**			
Abstract	2	See the PRISMA 2020 for Abstracts checklist.	Abstract
**INTRODUCTION**			
Rationale	3	Critical limitations of conventional SAR frameworks and the need for physicochemical reinterpretation in polymeric systems	Section 1. Introduction
Objectives	4	Objective to reinterpret SAR through physicochemical mediation and context-dependent functionality, and to establish a mechanistic–structural interpretive framework	Section 1. Introduction (final paragraphs before Figure 1)
**METHODS**			
Eligibility criteria	5	Interpretability-oriented eligibility criteria focusing on structural characterization, biological activity domains, and mechanistic relevance.	Section 2.2. Eligibility principles
Information sources	6	Scopus and Web of Science Core Collection databases, supplemented by reference list screening.	Section 2.1. Search logic and role of PRISMA
Search strategy	7	Keyword-based search strategy with predefined terms; full search strings are provided in the Supplementary Material.	Section 2.1. Search logic and role of PRISMA; Supplementary Material
Selection process	8	Title/abstract and full-text screening followed predefined eligibility criteria (Section 2.2). Both stages were performed independently and in parallel by two reviewers (Tran Thi Thu Suong and Hoang Ngoc Cuong); disagreements were resolved by discussion between the two until consensus, with no third-party adjudicator required. Post-inclusion evidence appraisal applied a pre-defined three-tier decision rule (Tier A/B/C; Section 2.3), replacing the previous qualitative 'conceptual alignment' approach.	Section 2.1 and Section 2.2
Data collection process	9	Structured data extraction focusing on structural characteristics, physicochemical mediators, and functional outcomes for each included study, followed by the Tier A/B/C evidence-integration classification (Section 2.3). The extracted data for all 26 included studies were manually verified independently and in parallel by two reviewers (Tran Thi Thu Suong and Hoang Ngoc Cuong); disagreements were resolved by discussion until consensus.	Section 2.3. Evidence-integration tiers (post-inclusion appraisal)
Data items	10a	Biological activity outcomes including antioxidant, anti-inflammatory, and prebiotic effects.	Section 2.2. Eligibility principles

Section and Topic	Item #	Checklist item	Location where item is reported
	10b	Structural attributes (molecular weight, branching architecture, conformation), methodological characteristics, and experimental context.	Section 2.2. Eligibility principles; Table 2
Study risk of bias assessment	11	A structured qualitative interpretive appraisal framework was applied instead of conventional risk-of-bias tools, focusing on structural confirmation, molar-mass reliability, branching resolution, conformation evidence, assay comparability, and co-variation control (the previously used 'context alignment' criterion was removed to avoid circular reasoning).	Section 2.3. Evidence-integration tiers (post-inclusion appraisal); Table 1
Effect measures	12	No quantitative effect measures were used; outcomes were interpreted qualitatively based on reported bioactivity trends.	Section 2.4. Rationale for non-meta-analytic synthesis
Synthesis methods	13a	Mechanistic–interpretive qualitative synthesis integrating structural resolution, physicochemical mediation, and experimental context.	Section 2.4. Rationale for non-meta-analytic synthesis
	13b	Qualitative categorization and structured synthesis using evidence appraisal matrix (Table 1) and domain-specific tables.	Section 2.3; Table 1
	13c	Results were tabulated and structured using an interpretive appraisal matrix (Table 1) and domain-specific synthesis tables (Tables 2–3), complemented by qualitative synthesis across thematic sections.	Table 1; Table 2; Table 3; Sections 3.2–3.3
	13d	A mechanistic–interpretive qualitative synthesis was performed, integrating structural attributes, physicochemical behavior, and experimental context to explain heterogeneity and avoid reductionist structure–function inference.	Section 2.4. Rationale for non-meta-analytic synthesis
	13e	Heterogeneity was explored qualitatively by examining variations in structural resolution, physicochemical behavior, and experimental context across studies.	Sections 2.3–2.4; Sections 3.4–3.8
	13f	A sensitivity analysis was performed by repeating the principal synthesis after excluding Tier C studies; principal conclusions were retained, with the exception of the film-antioxidant observation in Section 3.8, which rests on a single lower-confidence study.	Section 2.4; Supplementary Table S5
Reporting bias assessment	14	Reporting bias was considered qualitatively through comprehensive database searching, iterative screening, and reference list cross-checking.	Section 2.1. Search logic and role of PRISMA
Certainty assessment	15	Formal certainty assessment was not conducted due to the qualitative and interpretive nature of the synthesis.	Section 2.3 and Section 2.4

PRISMA 2020 Checklist

Section and Topic	Item #	Checklist item	Location where item is reported
**RESULTS**			
Study selection	16a	Study selection results are presented using a PRISMA 2020 flow diagram (Figure 2), summarizing identification, screening, eligibility, and inclusion processes.	Figure 2; Section 3.2
	16b	Studies excluded during screening and eligibility assessment are summarized in the PRISMA flow diagram based on predefined criteria.	Figure 2; Section 3.2
Study characteristics	17	Study characteristics, including structural attributes, biological activity domains, and experimental context, are summarized using an interpretive appraisal matrix (Table 1) and domain-specific synthesis tables.	Table 1; Table 2; Sections 3.1–3.3
Risk of bias in studies	18	Risk of bias was not assessed using conventional tools; instead, a structured qualitative interpretive appraisal framework was applied, focusing on structural confirmation, molar-mass reliability, branching resolution, conformation evidence, assay comparability, and co-variation control (the previously used 'context alignment' criterion was removed to avoid circular reasoning).	Section 2.3; Table 1
Results of individual studies	19	Results of individual studies are summarized descriptively through structured tables, including key structural parameters and reported bioactivities across studies.	Table 1; Table 2; Table 3
Results of syntheses	20a	Synthesized results are presented through qualitative integration of structural characteristics, physicochemical behavior, and biological activity across thematic domains.	Sections 3.4–3.8; Table 3
	20b	No statistical synthesis or meta-analysis was conducted due to heterogeneity in structural characterization and experimental conditions.	Section 2.4
	20c	Heterogeneity is explored qualitatively through variations in molecular structure, physicochemical behavior, and experimental context across studies.	Sections 3.4–3.8
	20d	A sensitivity analysis was performed by repeating the principal qualitative synthesis after excluding the five Tier C studies (restricted corpus, n = 21); principal conclusions were retained, with one exception (the film-antioxidant observation, Section 3.8) explicitly flagged as resting on a single lower-confidence study. Full reproducible comparison in Supplementary Table S5.	Section 2.4; Supplementary Table S5
Reporting biases	21	Reporting bias was considered qualitatively through comprehensive literature search, screening, and cross-referencing procedures.	Section 2.1
Certainty of evidence	22	Formal certainty assessment was not conducted due to the qualitative and interpretive nature of the synthesis.	Section 2.3; Section 2.4
**DISCUSSION**			
Discussion	23a	The findings are interpreted through a mechanistic–contextual framework integrating structural attributes, physicochemical mediation, and biological functionality.	Sections 4.1–4.9
	23b	Limitations of the included evidence are discussed in terms of structural heterogeneity, methodological variability, and insufficient physicochemical characterization.	Sections 4.1, 4.5, 4.7
	23c	Limitations of the review process include the qualitative interpretive approach, absence of quantitative synthesis, and reliance on available reporting in primary studies.	Sections 2.4 and 4.5
	23d	Implications for research and application are discussed in terms of improving SAR interpretability, integrating physicochemical frameworks, and enhancing reproducibility in food and biological systems.	Sections 4.6–4.9; Conclusions
**OTHER INFORMATION**			
Registration and	24a	This review was not prospectively registered, and no formal review protocol was prepared.	Section 2.1

PRISMA 2020 Checklist

Section and Topic	Item #	Checklist item	Location where item is reported
protocol	24b	No protocol was prepared; therefore, no protocol is available.	Section 2.1
	24c	Describe and explain any amendments to information provided at registration or in the protocol.	Not applicable.
Support	25	No specific funding was received for this work.	Acknowledgements
Competing interests	26	Competing interests are declared in the Conflicts of interest statement within the manuscript and, where required, in a separate declaration document.	Conflicts of interest statement
Availability of data, code and other materials	27	Data supporting the findings are provided as Supplementary Data Files S1–S4: search history summary; full-text exclusion list with reasons (n = 619); PRISMA 2020 accounting; and the study-level extraction dataset (n = 26 studies). Raw database export files are archived by the corresponding author and available upon reasonable request.	Data availability statement; Supplementary Information

From: Page MJ, McKenzie JE, Bossuyt PM, Boutron I, Hoffmann TC, Mulrow CD, et al. The PRISMA 2020 statement: an updated guideline for reporting systematic reviews. BMJ 2021;372:n71. doi: 10.1136/bmj.n71. This work is licensed under CC BY 4.0. To view a copy of this license, visit https://creativecommons.org/licenses/by/4.0/
