# Supplementary material for: A systematic review of structure–activity relationships in bacterial levan: physicochemical mediation and context-dependent functionality
Source: RSC Adv. 2026 Oct 5;16(46):59022–44. doi: 10.1039/d6ra04924a (PMC13636291; doi:10.1039/d6ra04924a)
Supplement: Supplementary file 2 [file RA-016-D6RA04924A-s002.pdf]

Supplementary Information (SI) for RSC Advances.
This journal is © The Royal Society of Chemistry

Supplementary Materials

S1. Full Search Strategy (Scopus & Web of Science)

Three progressively refined search strings (Tier 1–3, from broad sensitivity-maximizing terms to structure–activity-relationship-specific terms) were executed separately in each database, applied to the Title, Abstract, and Keywords fields (Scopus: TITLE-ABS-KEY; Web of Science: TS=). An English-language restriction was applied at the platform level in both databases (Scopus: Language; Web of Science: Language); document type and publication year were not restricted at search time and were instead enforced during title/abstract and full-text screening (Sections 2.1–2.2 of the main text).

Tier 1 was designed to maximize sensitivity and capture the broad landscape of microbial levan production. Scopus: TITLE-ABS-KEY((“levan” OR “fructan type exopolysaccharide” OR “fructan” OR “exopolysaccharide” OR “levansucrase”) AND (“microbial” OR “bacterial” OR “fermentation” OR “levan producing bacteria” OR “levanogenic strains”)). Web of Science: TS=((“levan” OR “fructan type exopolysaccharide” OR “fructan” OR “exopolysaccharide” OR “levansucrase”) AND (“microbial” OR “bacterial” OR “fermentation” OR “levan producing bacteria” OR “levanogenic strains”)).

Tier 2 applied functional and structural refinement to intersect production terms with specific descriptors. Scopus: TITLE-ABS-KEY((“levan” OR “fructan type exopolysaccharide” OR “fructan” OR “exopolysaccharide”) AND (“molecular weight” OR “branching” OR “glycosidic linkage” OR “structural characterization”) AND (“antioxidant” OR “anti-inflammatory” OR “prebiotic”)). Web of Science: TS=((“levan” OR “fructan type exopolysaccharide” OR “fructan” OR “exopolysaccharide”)

AND (“molecular weight” OR “branching” OR “glycosidic linkage” OR “structural characterization”) AND (“antioxidant” OR “anti-inflammatory” OR “prebiotic”)). This tier ensured the retrieval of studies reporting both structural parameters and biological outcomes necessary for SAR analysis.

Tier 3 targeted high resolution retrieval of studies explicitly addressing structure–activity correlations. Scopus: TITLE-ABS-KEY((“levan” OR “fructan type exopolysaccharide”) AND (“molecular weight” OR “degree of branching” OR “glycosidic linkage”) AND (“antioxidant” OR “anti-inflammatory” OR “prebiotic”) AND (“structure–activity relationship” OR “structure–function relationship” OR “structure–function correlation”)). Web of Science: TS=((“levan” OR “fructan type exopolysaccharide”) AND (“molecular weight” OR “degree of branching” OR “glycosidic linkage”) AND (“antioxidant” OR “anti-inflammatory” OR “prebiotic”) AND (“structure–activity relationship” OR “structure–function relationship” OR “structure–function correlation”)).

Records retrieved by the three tiers within each database were exported and merged with the results of the other database into a single pooled corpus (Scopus: n = 11,103, exported across three batches on 16 August 2025; Web of Science Core Collection: n = 7,110, exported across eight batches on 16 August 2025; combined total n = 18,213). Deduplication was performed once across the full pooled corpus using a hierarchical exact-match procedure implemented in R: records were matched first by DOI, then, for records lacking a usable DOI, by normalized title and publication year, and finally by normalized title, first author, and publication year. This procedure removed 5,385 duplicate records (5,361 by DOI

match, 24 by title-and-year match, 0 by title-author-year match), leaving 12,828 unique records for title/abstract screening.

Reference lists of the 26 included studies and of relevant review articles identified during screening were additionally searched by hand. This step did not identify any eligible study beyond those already retrieved through the database searches (0 additional records).

S2. PRISMA 2020 Flow Description

Initial records identified: 18,213

Duplicates removed: 5,385

Records screened: 12,828

Records excluded (title/abstract): 12,122

Reports sought for retrieval: 706

Reports not retrieved: 61

Reports assessed for eligibility: 645

Reports excluded (full-text, with reasons): 619

Studies included: 26

Search dates and screening workflow: Scopus was searched on 16 August 2025 (10:03-10:56 UTC) and Web of Science Core Collection on 16 August 2025 (11:22-11:27 UTC). Title/abstract screening, full-

text screening, and verification of data extraction for all 26 included studies were each performed independently and in parallel by two reviewers (Tran Thi Thu Suong and Hoang Ngoc Cuong); disagreements were resolved by discussion between the two reviewers until consensus was reached, with no third-party adjudicator required. Reference-list screening of the included studies and relevant reviews (Section S1) did not identify additional eligible records beyond those retrieved through the database searches.

S3. Evidence-Integration Tiers (Tier A / B / C)

Post-inclusion appraisal used three evidence-integration tiers, assigned from observable, pre-defined fields (Table S1): whether polymer structure, a candidate physicochemical mediator, and a functional outcome were measured within the same study, and whether a relevant comparator/control and major structural co-variation were controlled or explicitly modelled.

Tier A - direct within-study support:

Structure, mediator and function measured in the same system; relevant comparator/control; co-variation controlled or explicitly modelled.

May support a specific mediation hypothesis; does not establish causality unless mediation was experimentally tested.

Tier B - indirect/triangulated support:

Structure and function measured, but mediator unmeasured or co-variation incompletely controlled.

Supports a constrained association or hypothesis; wording remains non-causal.

Tier C - contextual mapping only:

Minimal eligible structural measurement, unreliable/unclear descriptors, heterogeneous comparison, or insufficient co-variation control.

Used to map reporting and heterogeneity; excluded from positive mechanistic inference.

Decision rule:

Tier assignment follows the criteria in Table S1 (structural confirmation, molar-mass reliability, branching resolution, conformation evidence, assay comparability, co-variation control). The previously used 'context alignment' dimension has been removed because it risked circular reasoning between structural attributes and reported outcomes.

A sensitivity analysis repeating the principal synthesis after excluding Tier C studies is reported in Section S6.

S4. Dataset Description (26 Studies)

The full study-level extraction dataset, the PRISMA 2020 accounting, the complete list of 619 full-text exclusions with reasons, and the search history summary are provided as Supplementary Data Files S1–S4 (see Data availability statement in the main text). Raw database export files (Scopus and Web of Science) are archived by the corresponding author and available upon reasonable request.

S5. Rationale for Non-Meta-Analysis

Meta-analysis was not conducted due to substantial heterogeneity in structural characterization methods, incomplete reporting of key parameters, and variability in experimental systems, which precluded meaningful quantitative synthesis.

S6. Limitations and Sensitivity Considerations

A sensitivity analysis was performed by repeating the principal synthesis after excluding the five Tier C studies. Principal conclusions were retained across regimes, with one exception: the film-antioxidant observation reported for material/film performance (main text, Section 3.8) rests on a single Tier C study and should be interpreted with corresponding caution. The full reproducible comparison of the full corpus (n = 26) against the Tier C-excluded restricted corpus (n = 21) is given in Supplementary Table S5.

Supplementary Tables

Supplementary Table S1. Evidence-appraisal criteria (coding scheme for Tier A/B/C assignment)

**Dimension**	**High**	**Moderate**	**Low**	**None / Proxy**

**Structural confirmation**	Full NMR (1D/2D) + SEC/MALLS	Partial (FTIR + NMR or SEC)	Indirect (FTIR only)	Not reported
**MW reliability**	Absolute (SEC-MALLS/TDA)	Relative (SEC vs standards)	Viscometry/DLS	Not reliable
**Branching resolution**	Quantitative (methylation + NMR)	Qualitative indication	Mentioned but unclear	Not reported
**Conformation evidence**	Direct (SAXS, rheology, etc.)	Indirect validated	Proxy (Congo red, SEM)	None
**Assay comparability**	Standardized assays	Comparable but varied	Heterogeneous endpoints	Not comparable
**Co-variation control**	Explicit control (fractionation, design)	Partial	Minimal	None

Supplementary Table S2. Structural characteristics of bacterial levan with interpretability constraints for SAR inference (n = 26)

NR: Not reported. MW: molecular weight (kDa unless otherwise stated).

**Study ID**	**Producing organism**	**Strain/isolate**	**Levan confirmation (evidence)**	**Molecular weight (kDa) / apparent MW (kDa)**	**MW method**	**Linkage pattern**	**Branching degree/index**	**Conformation / morphology / solubility**	**Structural characterization methods**	**Structural interpretability**	**MW interpretability**	**Evidence-integration tier (finalized; see Table 1)**
Included_01_k_2005	*Zymomonas mobilis*	ZM1 (ATCC 10988)	**Measured** (NMR + SEC)	2250	SEC/GPC (LS + viscometer)	β-(2→6) main; β-(2→1) branches	NR	Self-assembled nanoparticles (TEM/particle size)	13C-NMR; TEM; particle size analysis	High	Absolute	Tier B
Included_02_am_2012	*Bacillus subtilis*	NRC1aza	**Inferred/limited** (hydrolysis products; no NMR reported)	85.23; 31.95	Viscometry	β-(2→6) reported/inferred	NR	NR	Paper chromatography; HPLC; acid hydrolysis	Low	Proxy	Tier C
Included_03_m_2013	*Bacillus licheniformis*	FRI MY-55	**Inferred/unclear** (structural method NR)	13.45 (reported range 13.45→2.96)	NR	β-(2→6) cited generally	NR	NR	NR	Low	Not reliable	Tier C
Included_04_mat_2013	*Bacillus subtilis*	M (honey isolate)	**Measured** (NMR + FTIR)	21.8; 13.118; 9.53 (crude NR)	Viscometry	β-(2→6) main	NR	NR	1H/13C NMR (incl. 2D); FTIR; chromatography	Moderate	Proxy	Tier B

Included_05_r_2015	*Acetobacter xylinum*	NCIM 2526	**Measured** (NMR + FTIR)	NR	NR	β-(2→6) main; β-(2→1) branches	Qualitative (branched; not quantified)	NR	FTIR; 1H/13C NMR; TGA; HPLC/sugar analysis	Moderate	Not reliable	Tier B
Included_06_a_2019	*Bacillus subtilis* (enzyme source)	DSM 347	**Measured** (SEC-TDA)	29.8; 2.3	GPC-SEC TDA (absolute mass)	β-(2→6) main	NR	LOS fraction/short chains after depolymerization (functional morphology NR)	GPC (TDA/CC); UHPLC-MS (derivatives)	High	Absolute	Tier B
Included_07_m_2019	*Bacillus subtilis* natto	KB1	**Measured** (NMR + SEC)	37.62	SEC/GPC (triple detection)	β-(2→6) main; β-(2→1) branches (signals weak)	Low/qualitative (weak branching signals)	NR	FTIR; 1H/13C NMR; SEM; TGA; GPC	Moderate	Relative	Tier B
Included_08_a_2020	*Bacillus subtilis*	AF17	**Measured** (NMR + methylation + SEC-TDA)	20000	HP-SEC-TDA	β-(2→6) main; β-(2→1) branches	Low (linear:branched ≈ 20:1; ~5%)	Aggregation checked across pH (reported)	Methylation (GC-MS); 1D/2D NMR; SEC-TDA	High	Absolute	Tier B
Included_09_f_2020	*Bacillus megaterium*	PFY-147	**Measured** (NMR + SEC)	1878	GPC	β-(2→6) main	NR	Surface morphology (SEM/AFM); porous/compact noted	FTIR; 1H/13C NMR (incl. 2D); SEM; AFM; HPLC	Moderate	Relative	Tier B
Included_10_tim_2020	*Bacillus* sp.	honey isolate	**Measured** (NMR + XRD/SEM)	21	Viscometry	β-(2→6) main	NR	Adhesive/film forming; morphology (SEM/XRD)	SEM; XRD; 1H/13C NMR; chromatography	Moderate	Proxy	Tier B

Included_11_sv_2021	*Bacillus* sp.	4A	**Limited** (FTIR + sugar profile; no MW)	NR	NR	NR (FTIR similarity)	NR	NR	FTIR; HPLC (monosaccharide after hydrolysis)	Low	Not reliable	Tier C
Included_12_thn_2021	*Bacillus velezensis*	VTX20	**Measured (partial)** (NMR confirms levan-rich EPS; MW NR)	NR	NR	β-(2→6) main	NR	High water solubility reported	1H/13C (HMBC) NMR; HPLC; genome mining	Moderate	Not reliable	Tier B
Included_13_g_2022	*Pseudomonas mandelii*	NR	**Limited** (FTIR-based; MW NR)	NR	NR	β-(2→6) cited generally	NR	Film morphology (SEM); "nano-levan" label but MW NR	FTIR; SEM; TGA	Low	Not reliable	Tier C
Included_14_b_2023	*Bacillus subtilis*	MTCC 441	**Measured** (NMR + SEC)	2831.2	GPC	β-(2→6) main	NR	NR	1H/13C NMR; GPC	Moderate	Relative	Tier B
Included_15_oe_2023	*Halomonas elongata*	153B	**Measured** (NMR + MALLS-GPC)	3260	MALLS-GPC	β-(2→6) main (FTIR/NMR similarity)	NR	NR	FTIR; 1H NMR; MALLS-GPC	High	Absolute	Tier B
Included_16_s_2023	*Bacillus* sp.	EPS003	**Measured (partial)** (1H NMR + FTIR; MW NR)	NR	NR	β-(2→6) linkage	NR	Morphology/porosity (SEM/XRD/EDAX)	TLC; FTIR; 1H NMR; FE-SEM; XRD; EDAX; TGA	Moderate	Not reliable	Tier B
Included_17_sa_2023	*Pseudomonas aeruginosa*	HI1 (LevAE/others)	**Measured (partial)** (NMR/FTIR; MW NR)	NR	NR	β-(2→6) main	NR	NR	TLC; HPLC; FTIR; 1H/13C NMR	Moderate	Not reliable	Tier B
Included_18_ym_2023	*Enterococcus faecium*	MC-5	**Measured (partial)** (NMR/FTIR;	24	SEC (dextran standards;	β-(2→6) units	NR	Congo red test (conformation reported); film	HPLC; NMR; SEM; Congo red; XRD; FTIR	Moderate	Relative	Tier B

			SEC with standards)		reported)			morphology (SEM/XRD)				
Included_19 _j_2024	*Bacillus subtilis*	PR-C18	**Measured** (NMR + SEC)	34	SEC/GPC (standards)	β-(2→6) main	NR	Porous morphology (SEM/EDX)	FTIR; 1H/13C NMR; SEM; EDX	Moderate	Relative	Tier B
Included_20 _k_2024	*Bacillus megaterium*	KM3	**Limited/partial** (FTIR + Congo red; MW NR)	NR	NR	β-(2→6) inferred	NR	Triple-helix reported (Congo red)	HPLC; FTIR; XRD; DSC/TGA; Congo red	Low	Not reliable	Tier C
Included_21 _vs_2024	*Paenibacillus polymyxa*	88A	**Measured** (2D NMR + HPAEC-PAD + SEC)	1900	HPGPC (standards)	β-(2→6) main	Slightly branched (qualitative)	SEM/AFM morphology; rheology reported	HPAEC-PAD; FTIR/DRIFT; 1D/2D NMR; SEM; AFM; TGA/DSC; rheology	High	Relative	Tier B
Included_22 _wa_2024	*Bacillus megaterium* + *Rhizobium* sp. (coculture)	OP315322/OP363928	**Measured (partial)** (NMR/FTIR; MW via viscometry)	11.5	Viscometry	β-(2→6) main	NR	NR	TLC; FTIR; 1H/13C NMR	Moderate	Proxy	Tier B
Included_23 _h_2025	*Bacillus amyloliquefaciens*	JM033	**Measured** (methylation + 2D NMR + SEC-MALLS)	17.6	SEC-MALLS-RI	β-(2→6) backbone + β-(2→1) branches (hybrid)	Branched (reported; not a single % value)	Spherical configuration reported	HPAEC-PAD; FTIR; methylation (GC-MS); 1D/2D NMR; SEC-MALLS	High	Absolute	Tier B
Included_24 _j_2025	*Bacillus licheniformis*	SFLV-ZM107	**Measured** (SEC/MALLS + FTIR)	5.655; 15.76	GPC-MALLS	β-(2→6) main	NR	Morphology (SEM/AFM); rheology reported	FTIR; GPC; SEM; AFM; TGA; rheology	High	Absolute	Tier A

Included_25_nny_2025	*Gluconobacter cerinus*	UELBM 11	**Measured** (methylation + NMR + HPSEC-MALLS)	878	HPSEC-MALLS	β-(2→6) main; β-(2→1) branches	Branched (5.87% branching points reported)	Microporous morphology (SEM)	Methylation (GC-MS); FTIR; 1H/13C NMR; SEM; TGA/DTG	High	Absolute	Tier B
Included_26_r_2025	*Bacillus siamensis*	KKSB4 (and other strains in study)	**Measured** (NMR/FTIR)	**Apparent MW (hydrodynamic, DLS-derived):** 61,000; 220,000; 85,000; 69,000	**DLS/Zetasizer** (hydrodynamic sizing; reports apparent MW derived from hydrodynamic size, not absolute molar mass)	β-(2→6) main	NR	Porous/complex morphology implied; DSC reported	TLC; FTIR; 1H/13C NMR; DSC	Moderate	Proxy	Tier B

Note: Molecular weight (MW) values represent weight average molar mass (kDa) unless specified as apparent values derived from hydrodynamic size. In accordance with polymer science standards, higher structural confidence is attributed to studies utilizing absolute detection methods (e.g., SEC–MALLS, SEC–TDA) compared to relative methods (e.g., viscometry or calibration with standards). Taxonomic names are presented in accordance with international nomenclature standards. Linkage patterns and branching features follow the notation β-(2→6) for the levan backbone and β-(2→1) for branching residues. Abbreviations: NR, not reported in the original study; DLS, dynamic light scattering; FTIR, Fourier-transform infrared spectroscopy; GPC/SEC, gel permeation/size-exclusion chromatography; MALLS, multi-angle light scattering; NMR, nuclear magnetic resonance spectroscopy; TDA, triple detector analysis; TEM, transmission electron microscopy. 'Structural interpretability' and 'MW interpretability' report the per-dimension component ratings defined in Supplementary Table S1; these feed into, and are superseded by, the single integrated Evidence-integration tier (Tier A/B/C, Section S3) reported in the final column — they are descriptive inputs, not a parallel or additional scoring system.

Supplementary Table S3. Property-mediated SAR evidence linking levan conformation, solubility, and aggregation to bioactivity under context-dependent physicochemical constraints

Conformation / solubility / aggregation descriptor (proxy)	Endpoint context	Activity domain(s)	Direction of effect	Evidence consistency	Studies contributing	Notes (mechanistic interpretation + confounding)	Physicochemical property	Mechanistic role
**Self-assembled nanoparticles (170–300 nm; amphiphilic self-assembly)**	Ex vivo / in vivo topical + cell models	Anti-inflammatory	↑	**Weak**	**Included_01**	Single study evidence. The reported effect was attributed to nanoscale dispersion/penetration despite HMW (2,250 kDa). Not comparable to radical scavenging assays.	Surface area / dispersion	Enhances interface interaction with biological barriers
**Aggregation state explicitly assessed (pH dependent checks; "HMW not due to aggregation")**	Chemical assays (solution) + structural validation	Antioxidant (interpretability of MW/activity)	↔ (control)	**Weak**	**Included_08**	This was treated as methodological support for interpreting the reported MW as non-aggregated. Does not directly establish conformation-driven bioactivity.	Aggregation stability	Ensures MW interpretation validity rather than direct activity
**Congo red assay indicates ordered conformation**	Chemical assays (solution)	Antioxidant	↑ (association)	**Weak–Moderate**	**Included_20; Included_18**	Proxy-based conformation assignment. Outcomes are antioxidant assays	Structural	Stabilizes polymer–radical

**(reported triple-helix)**						(DPPH/hydroxyl, etc.) but endpoints differ and one study is material/composite oriented. The association was attributed to increased stability/organization rather than direct radical scavenging.	organization	interaction environment
**Film forming / adhesive property (protective layer mechanism)**	In vivo gastric ulcer + in vitro adhesion	Anti-inflammatory and prebiotic	↑ (context dependent)	**Moderate (context specific)**	**Included_10**	Strong within study mechanistic logic (protective coating; reduced NF-κB/COX-2) but limited cross study replication. Represents "property→function" mechanism, not chemical antioxidant scavenging.	Matrix formation / adhesion	Forms protective interface layer
**Porous / compact microstructure (SEM/AFM) linked to interface/water holding**	Chemical assays (solution)	Antioxidant	↑ (context dependent)	**Moderate**	**Included_09; Included_21; Included_25**	Multiple studies report SEM/AFM morphology and antioxidant activity. However, morphology co-varies with MW and composition (e.g., non-carbohydrate substituents reported in Included_09; sulfate groups in	Surface area / porosity	Enhances interfacial exposure to reactive species

						Included_21). Treat as correlational.		
**Porous morphology / high water retention discussed as activity relevant**	Chemical assays + biofilm/prebiotic (in vitro)	Antioxidant + prebiotic (supportive)	↑ (mainly antioxidant)	**Weak–Moderate**	**Included_19**	SEM porous structure + DPPH antioxidant + prebiotic/antibiofilm. Multi-endpoint study; mechanism discussion is plausible but not replicated across matched assays.	Hydration / porosity	Supports interaction and retention of reactants/ microbes
**Porosity / hydrophilic matrix implied by FE-SEM/XRD/EDAX; high water-holding capacity**	Chemical assays (solution) + cytotoxicity	Antioxidant	↑	**Weak**	**Included_16**	MW not reported; structure to property claims rely on general porosity/hydrophilicity. Useful supportive evidence but low comparability.	Hydrophilicity / porosity	Improves diffusion and interaction
**Composite / material film matrix (levan films; microstructure emphasized)**	Material-based assays (films/composites)	Antioxidant (film DPPH)	↑ (material endpoint)	**Weak**	**Included_13; Included_18**	Evidence pertains to **matrix-mediated performance** (film antioxidant) and additive effects (e.g., composites). Should not be interpreted as intrinsic biological antioxidant activity of levan in vivo/cell systems.	Matrix structure	Enables performance via matrix-mediated interaction
**Rheology/viscosity and chain entanglement (reported; higher**	Fermentation / growth assays (in	Prebiotic utilization /	↓ (for higher viscosity/entanglement)	**Moderate**	**Included_24 (LOS vs LMW fractions); Included_06**	Included_24 provides direct comparative framework with rheology + prebiotic	Viscosity /	Limits enzymatic

**viscosity/complexity)**	vitro) + rheology	fermentation efficiency			**(supporting size/compactness rationale)**	outcome: higher viscosity/complexity aligns with reduced growth promotion vs LMW fraction. Included_06 supports "size/compactness limits utilization" (though rheology not explicitly measured).	entanglement	accessibility
**Emulsifying/hydrogel forming behavior (structural organization in food matrix)**	Simulated GIT / in vitro functional system	Prebiotic (hydrolyzed form better)	↑ (after hydrolysis)	**Weak**	**Included_11**	Study focuses on emulsifying hydrogels; prebiotic improvement reported for hydrolyzed form (implies accessibility rather than a defined conformation). Structural descriptors are indirect; MW not reported.	Hydration / accessibility	Improves enzymatic and microbial access
**UHMW reported by Zetasizer (method limitation) + WHC/OHC "complex/porous" claims**	Chemical assays + prebiotic score (in vitro)	Antioxidant and prebiotic	↔ / context dependent	**Weak**	**Included_26 (KKSB4 and other strains; DLS derived apparent MW)**	Evidence is heterogeneous with strong **methodological uncertainty** (Zetasizer is not a standard MW determination method for polymers). Mixed prebiotic outcomes despite very high "MW", suggesting conformation/accessibility or measurement	Aggregation + measurement artefact	Confounded interpretation due to method limitation

						artifact. Should be flagged as exploratory only.		

Note: Directional SAR trends (↑, ↓, ↔) indicate the most frequently reported qualitative associations within the stated assay context and do not imply causality or effect size optimization. Activity domains are strictly limited to the review's scope (antioxidant, anti-inflammatory, and prebiotic effects). Evidence consistency is graded based on the number of concordant studies and the degree of methodological heterogeneity: Strong (≥3 concordant studies); Moderate (2–3 studies); and Weak (single study or highly heterogeneous proxies). Supramolecular descriptors (e.g., triple-helix, porosity) are predominantly inferred from indirect proxies (Congo red, SEM/AFM) rather than standardized conformational measurements. Where antioxidant or anti-inflammatory outcomes were assessed in material-based systems (films or composites), they were interpreted as matrix-mediated performance to elucidate structural influence on bioactivity. Abbreviations: AFM, atomic force microscopy; DLS, dynamic light scattering; SEM, scanning electron microscopy; UHMW, ultra high molecular weight; XRD, X-ray diffraction.

Supplementary Table S4. Exploratory SAR evidence from chemically modified levan under confounding structural and physicochemical changes

Study ID	***Modification type***	***Activity***	***Outcome (original)***	***Primary modification effect***	***Confounding factors***	***Permissible interpretation***
Included_02	*Sulfated levan*	*Antioxidant*	*Enhanced activity vs native*	*Charge introduction (–SO₃⁻)*	*MW change; conformation shift; solubility alteration; purity*	*Exploratory only*
Included_02	*Sulfated levan*	*Cell-based*	*Increased cytoprotection*	*Increased interaction with biological targets*	*Same as above + assay sensitivity*	*Context-specific*

Note: Directional association (↑) reflects a qualitative increase in bioactivity relative to the native polymer within the same experimental framework. Evidence is classified as Limited due to reliance on a single study comparison. The SAR interpretation accounts for potential confounding by unresolved molecular weight shifts and purity changes during the derivatization process. Abbreviations: DS, degree of substitution; MW, molecular weight; DPPH, 2,2-diphenyl-1-picrylhydrazyl.

Supplementary Table S5. Sensitivity analysis: full corpus (n = 26) versus Tier C-excluded restricted corpus (n = 21)

**Regime / domain**	**Full corpus, supporting studies (n)**	**Of which Tier C (Study ID)**	**Restricted corpus (Tier C excluded), supporting studies (n)**	**Conclusion after Tier C exclusion**

Chemical antioxidant (chemical assays)	19	3 (Included_02, Included_13, Included_20)	16	Retained — higher apparent MW vs. higher apparent radical-scavenging activity remains supported; causal/intrinsic-reactivity interpretation was never drawn (Section 3.4).
Cell-based inflammatory	6	0	6	Retained — unaffected; includes the review's only mechanistically modelled pathway (NF-κB/COX-2).
Microbial fermentation / prebiotic	9	1 (Included_11)	8	Retained — unaffected; the lower-MW-enhances-utilization trend remains anchored by the review's sole Tier A study (Included_24).
Gastrointestinal-protection	2	0	2	Retained — unaffected.
Material / film performance (general)	3	1 (Included_13)	2	Retained for the general film-performance observation (2 Tier B studies remain).
□ Film-antioxidant sub-claim (Section 3.8)	1	1 (Included_13)	0	Weakened — no longer supported once its sole underlying study is excluded; already flagged in the main text as resting on a single lower-confidence (Tier C) study.
Regime not clearly assignable (contextual mapping only)	2	1 (Included_03)	1	Not applicable — descriptive/heterogeneity mapping only, per the permitted use of Tier C evidence (Section 2.3); not used to support a positive conclusion in either corpus.

Note: Full corpus n = 26 (1 Tier A + 20 Tier B + 5 Tier C). Restricted corpus n = 21 excludes the five Tier C studies (Included_02, Included_03, Included_11, Included_13, Included_20; Table 1). Study counts per regime include multi-regime studies once in each applicable regime, consistent with Section 3.2 of the main text; totals therefore exceed 26 when summed across regimes. Re-synthesis under the restricted corpus used the same qualitative, mechanistic-interpretive procedure described in Section 2.4/S5 (no re-weighting or statistical aggregation), applied to the 21 remaining studies. Tier

C evidence is not used to support any positive conclusion in either corpus (Section 2.3); its exclusion here is reported for transparency and reproducibility.

Restricted corpus (n = 21), full study list: Included_01, Included_04, Included_05, Included_06, Included_07, Included_08, Included_09, Included_10, Included_12, Included_14, Included_15, Included_16, Included_17, Included_18, Included_19, Included_21, Included_22, Included_23, Included_24, Included_25, Included_26.
